# Cognitive Control and Metacognition: The Effect of Interference, Working-Memory Load, and Shifting on Confidence in Response Correctness and Partial Error

**DOI:** 10.3390/brainsci16090975

**Published:** 2026-09-15

**Authors:** Patryk Guzda, Marta Siedlecka, Aleksandra Gruszka

**Affiliations:** 1 Doctoral School in the Social Sciences, Jagiellonian University, 31-010 Krakow, Poland; 2 Institute of Psychology, Jagiellonian University, 30-060 Krakow, Poland

**Keywords:** cognitive control, metacognition, confidence judgments, performance monitoring, partial errors, electromyography, Stroop interference, stimulus complexity, metacognitive efficiency

## Abstract

**Highlights:**

**What are the main findings?**

- Response conflict impaired performance and was associated with a small reduction in confidence in the correctness of completed responses.
- In Experiment 2, conflict increased partial-error occurrence. Greater incorrect-burst surface and longer correction time were associated with higher confidence that a partial error had occurred, although both associations were weaker during compound processing.

**What are the implications of the main findings?**

- Lower confidence on consecutive compound trials suggests sensitivity of confidence judgments to sustained processing demands.
- The relationship between response-related information and metacognitive judgments varies with current task demands.

**Abstract:**

Background/Objectives: Confidence is typically studied in simple perceptual decisions, although everyday decisions also require cognitive control and monitoring of action. Across two experiments, we examined whether response conflict, stimulus complexity, and sequence affect task performance and retrospective metacognitive judgments concerning different aspects of performance: confidence in response correctness and confidence that a covert partial error occurred. Methods: Participants performed a modified Stroop task in which they responded to word colour and, in compound trials, also reported the position of an accompanying line. In Experiment 1 (*N* = 66), participants rated confidence in the correctness of their responses. In Experiment 2 (*N* = 47), electromyography was used to identify covert partial errors, and participants rated confidence that a partial error had occurred. Results: Conflict reduced response accuracy, prolonged response times, and was associated with a small reduction in confidence in correct responses. In Experiment 1, the effect of sequence on confidence depended on stimulus complexity: confidence was higher in simple-repeated than in simple-switched trials, whereas the opposite pattern was observed for compound trials. In Experiment 2, partial errors occurred more frequently under conflict but less frequently in compound trials. Greater incorrect-burst surface and longer correction time were associated with higher partial-error confidence, and both relationships were weaker in compound trials. The association with incorrect-burst surface remained reliable after adjustment for response time. Exploratory analyses also showed lower metacognitive sensitivity under conflict and complexity and lower metacognitive efficiency in compound trials. Conclusions: Cognitive-control demands were associated with retrospective metacognitive judgments concerning both completed response outcomes and partial-error events occurring during response generation. Conflict was associated with a small reduction in confidence in correct responses, whereas partial-error confidence was associated with measurable characteristics of the partial-error event. The weaker relationship between EMG-derived response-process characteristics and confidence during compound processing suggests that current task demands affect how strongly internal response-related information is related to metacognitive judgments.

## 1. Introduction

Does confidence in our response change when a task becomes more challenging? Imagine that you are solving a simple test in which you must frequently inhibit an impulsive response or remember additional information. Would you be less certain of your answers, or more, when the task becomes more attentionally demanding?

In this article, we report two experiments examining how cognitive-control demands relate to task performance and metacognitive judgments. Specifically, we investigate three sources of task difficulty: response conflict, stimulus complexity, and sequence. In Experiment 1, participants evaluated the correctness of the completed colour-plus-line response sequence. In Experiment 2, participants evaluated whether a partial error had occurred during the preparation of the colour response. To manipulate task demands, we used a modified Stroop task in which participants responded to word colour and, on compound trials, also reported the position of an accompanying line.

In the present study, confidence refers to participants’ explicit judgments about their own preceding performance. Most of the theories aiming to explain confidence in perceptual decision tasks focus on how incoming sensory evidence informs confidence judgments (for a review, see, e.g., ref. [1]). The additional influences that are considered are usually different kinds of noise, although some models (i.e., ref. [2]) assume that one’s own actions also carry information used for computing confidence. Moreover, these theories refer to relatively simple, usually two-alternative decision tasks, in which the context is not relevant [3]. Outside simple laboratory decisions, perceptual judgments and decision-related actions are embedded in task goals and rules that determine which stimulus features are relevant, which responses are required, and how much information must be maintained, selected, or updated. Consequently, confidence and its correspondence with objective performance may depend not only on sensory evidence, but also on the cognitive demands imposed by the current task. More generally, cue-utilization accounts propose that metacognitive judgments are constructed from multiple internal and external cues rather than from direct access to objective performance itself [4]. Such judgments may be related to multiple aspects of the preceding task episode, including response timing, experienced difficulty, expectations, and information available after the response. Moreover, the degree of one’s confidence in response accuracy might serve as a signal of the need to adjust and regulate cognitive processing in accordance with task requirements. When confidence is low, individuals may hesitate, postpone further decisions, or even halt action entirely. This potential regulatory and control function of confidence resembles some aspects of cognitive control, such as performance monitoring [5].

The performance monitoring system is assumed to detect difficulties in information processing and signal the need for enhanced top-down control [6,7]. Performance monitoring has been linked to the detection of cognitive conflict and motor errors, including unintended actions [8] and premature responses [9], by a dedicated monitoring subsystem [10]. Importantly, performance monitoring is not limited to evaluating completed actions. It can also operate online while an action is being selected and prepared. Partial errors are a less studied but theoretically important example of this form of control. Partial errors occur when an incorrect motor activation is initiated and subsequently replaced by the correct response before producing an overt error [11,12,13]. Like overt errors, partial errors can elicit early error-related neural activity, such as the Ne, although this activity is typically smaller and is not necessarily followed by the Pe [12,14,15]. The Pe, in contrast, is more closely associated with conscious error awareness and, in detected partial-error trials, may emerge after the corrective response [12,16,17].

The same correct behavioural outcome can arise from different response trajectories. Although pure-correct and partial-error trials both end with a correct overt response, only the latter contain a verified preceding incorrect-hand activation. Partial errors therefore reveal aspects of response generation that remain invisible at the level of final behavioural accuracy and response time.

Metacognitive monitoring and performance monitoring may be interrelated; the brain may engage a set of monitoring subsystems that track performance indicators and integrate their signals as informational cues in shaping the experience of confidence. Thus, monitoring has been proposed as a candidate process linking executive functions with metacognition [18,19,20]. Findings from neural research indicate that neural activity associated with errors is correlated with reported decision confidence, perceived response inaccuracy, and error awareness [21,22,23,24]. For example, in a study specifically focused on the monitoring of one’s own motor-response execution, Ficarella et al. [12] showed that consciously detected partial errors are associated with stronger and more persistent incorrect motor activity than undetected partial errors. Culot et al. [25] further showed that partial-error detection and confidence increase with partial-error amplitude and correction time, and that motor signals have a stronger influence on sensorimotor confidence judgments when visual information is reduced. In another study, Gajdos et al. [26] showed that subthreshold motor activations were associated with higher perceptual confidence after controlling for accuracy and response-related covariates.

Cognitive conflict arises when task-relevant and task-irrelevant information activate incompatible response tendencies, as in an incongruent Stroop trial. Previous studies have shown that such conflict also influences confidence reports. For example, our previous study using a Stroop task showed that conflict reduces confidence in correct responses [27] and is associated with reduced conscious detection of motor errors [27,28]. Incongruent trials are also perceived as more difficult and increase the reported “urge to err” [27,29,30,31]. Perceived difficulty seems to be affected by different types of response conflicts; in a study by Desender and colleagues [32], participants rated trials in which a target stimulus was preceded by an incongruent cue as more difficult, as well as the trials requiring a response different from the previous one. Moreover, in another study, conflict adaptation was observed only after trials in which participants reported experiencing response conflict [33].

Finally, it has been shown that working-memory load also affects the level and accuracy (thus the relation to objective performance) of reported confidence, suggesting that confidence monitoring and reporting is resource demanding (see also [34]). Maniscalco and Lau [35] observed that the requirement to actively manipulate working-memory content impairs performance in the metacognitive task (confidence rating). Another study showed that under a high working-memory load, people are less prone to report uncertainty in their perceptual decisions, but that this effect disappears with training [36]. Lastly, in a study in which participants performed a visual short-term memory task, their response confidence was lower in trials in which an additional visual stimulus was presented [37].

In this study, we focus on three hypothetical cognitive-control functions, proposed by Miyake et al. [38]: inhibition, updating, and shifting. Inhibition allows the cognitive system to deal with interference, updating allows maintaining and actively manipulating the contents of working memory, and shifting involves the ability to switch between tasks or mental sets. For this purpose, we created a task inspired by those used in studies by Rietbergen et al. [39] and Sikora et al. [40], which were designed to vary demands associated with these three functions (note that no metacognitive judgments were collected in these studies). In the task used by Rietbergen et al. [39], the requirement of inhibiting was manipulated by presenting congruent and incongruent stimuli, the updating requirement was manipulated by presenting an additional line above or below the main stimulus, and the shifting requirement was manipulated by repeating or changing the type of trial. More control-demanding trials resulted in slower responses and an increased number of errors [39]. By creating similar conditions using a modified Stroop task, we aimed to measure the effects of conflict (increased demand for inhibiting), stimulus complexity (updating), and sequence (shifting) on reported confidence and its relation to objective measures of response accuracy and motor activity.

## 2. Experiment 1

In this experiment, we measured the effect of conflict, stimulus complexity, and sequence on task performance and on reported confidence in response correctness. We expected that more control-demanding conditions would result in higher behavioural cost: slower responses and lower accuracy [39]. We also assumed that the detection of cognitive and motor conflicts, errors, or increased task difficulty by the performance monitoring system would lower confidence in response accuracy. We thus hypothesized that increasing demands on each control function reduces confidence in one’s response; specifically, that individuals are less certain of their responses when they must inhibit a competitive response, when a trial requires maintaining more working-memory content, and when a stimulus type differs from the preceding one. Additionally, we estimated metacognitive sensitivity and efficiency (later referred to as measures of metacognitive monitoring). The former is presumed to measure how well confidence reports distinguish between correct and incorrect responses, while the latter presumably measures how efficiently information that led to task response is ‘‘used” by confidence judgments [41]. Our analyses concerning these measures were exploratory. On one hand, increased attentional and memory demands might decrease the relation of confidence reports to objective performance, if metacognitive monitoring is a resource-consuming task. On the other hand, if detecting increased task difficulty engages more cognitive resources, metacognitive measures could show more efficient metacognitive processes.

### 2.1. Materials and Methods

#### 2.1.1. Participants

Sixty-six volunteers participated in the study (52 women). The mean age of participants was 21.51 years (SD = 3.58, range 18–40). Participants provided informed consent and received the equivalent of 5.56 EUR. This study was approved by the Research Ethics Committee of the Institute of Psychology of Jagiellonian University (opinion no. KE/5_2023; 16 February 2023).

Since no prior study testing the effect of executive processes load on confidence was available to guide sample size determination, we estimated the minimal sample size required to observe the main effects of the executive component processes on response time (RT) based on Rietbergen et al. [39]. To ensure sufficient statistical power, we adopted a convenience sample of sixty-six participants, which exceeds the minimum required *N* = 22 calculated with G*Power (version 3.1.9.7) [42] (α = 0.05, Power = 0.95), with the expected effect size estimated based on the study reported by Rietbergen et al. [39].

#### 2.1.2. Materials

We used a modified version of the classic Stroop task [43], based on the design introduced by Rietbergen et al. [39]. Stimuli were presented as either simple or compound displays. Simple stimuli consisted only of a target colour word. Compound stimuli consisted of the target word together with a dashed line, presented either above or below the word.

The word stimulus was one of four Polish colour words corresponding to red, blue, green, and brown. Each word was displayed in one of four font colours: red, blue, green, or brown. All words were presented in lowercase and were approximately 2 cm high and 6 cm wide.

The task manipulated three factors: conflict, complexity, and sequence. Conflict referred to the relation between word meaning and font colour. In congruent trials, the word meaning matched the font colour; in conflict trials, the word meaning and font colour differed. Complexity referred to the amount of information present in the stimulus. In simple trials, participants were presented with only the colour word. In compound trials, they were presented with both the colour word and the additional dashed line. Sequence referred to whether the stimulus complexity repeated or changed from the preceding trial. Thus, a trial could be repeated, as in simple trials following simple trials or compound trials following compound trials, or switched, as in simple trials following compound trials or compound trials following simple trials.

These manipulations allowed us to estimate three behavioural effects. The conflict effect indexed the cost of resolving interference between word meaning and font colour, corresponding to the classic Stroop effect. The complexity effect indexed the additional demands associated with processing the compound stimulus and determining the presence and position of the dashed line. The sequence effect indexed the cost of switching between simple and compound trial types.

To reduce low-level stimulus repetitions, we used two alternating stimulus lists. Blue and green colour-word stimuli appeared on odd trials, whereas red and brown colour-word stimuli appeared on even trials. This ensured that stimulus features, such as word meaning and font colour, did not repeat across consecutive trials, allowing us to focus on changes related to higher-level cognitive adaptation. Each word colour appeared equally often in congruent and incongruent displays. For example, the list contained the same number of green words printed in green font and green words printed in blue font. This balancing was used to prevent participants from learning accidental stimulus-frequency regularities.

The stimulus lists were balanced across the main task conditions. The task included 448 trials in total: 112 simple-congruent trials, in which word meaning and font colour matched and no additional line was present; 112 compound-congruent trials, in which word meaning and font colour matched and an additional line was present; 112 simple-conflict trials, in which word meaning and font colour differed and no additional line was present; and 112 compound-conflict trials, in which word meaning and font colour differed and an additional line appeared either above or below the word.

#### 2.1.3. Procedure

The experiment was programmed in PsychoPy (version 2021.2.3) [44] and conducted in the Laboratory of the Department of Experimental Psychology at the Institute of Psychology, Jagiellonian University. Before the study, all participants provided written informed consent.

Participants were instructed to ignore the meaning of the word and respond to its font colour. After the colour response, they indicated whether the dashed line was absent, below the word, or above the word. At the end of each trial, they rated their confidence in the correctness of their response.

The experimental task was preceded by two training sessions. The first training session consisted of 8 neutral trials with letter strings, such as “OOOOO” or “XXXXX.” Its purpose was to familiarize participants with the colour-response procedure. Each trial began with a black screen presented for 300 ms, followed by a fixation point for 500 ms and another black screen for 300 ms. The target stimulus was then presented for 700 ms. After the target disappeared, a fixation point was shown, and 500 ms later, the response options appeared on the left and right sides of the screen, together with the fixation point in the centre. The response options were two colour words displayed in white, for example, “brown” on the left and “red” on the right. They remained on the screen until the participant responded, but no longer than 1.8 s.

In the second training session, participants responded first to the font colour and then to the line component. They indicated whether the dashed line was absent, below the word, or above the word using three keys: “SPACE” for no line, “K” for a line below the word, and “O” for a line above the word. The line-position response was made without time pressure. Participants did not provide confidence ratings during the training sessions.

The experimental session consisted of 14 blocks of 32 trials. Stimuli were presented in a random order. Each trial began with a black screen for 300 ms, followed by a fixation point for 500 ms and another black screen for 300 ms. The target colour-word stimulus was then presented for 500 ms. After the target disappeared, a fixation point was shown alone for 300 ms. Next, the current colour-response options appeared on the left and right sides of the screen, with the fixation point remaining in the centre. The response screen remained visible until the participant responded, but no longer than 1.6 s.

Participants responded to the font colour using two keys. The “Z” key was assigned to the left response and was pressed with the left index finger. The “M” key was assigned to the right response and was pressed with the right index finger. The mapping between the target colour and response side was balanced, such that the correct response appeared on the left side in 50% of trials and on the right side in the remaining 50%. If no colour response was given within the response window, the trial ended with the feedback “Too slow.”

**Figure 1:**
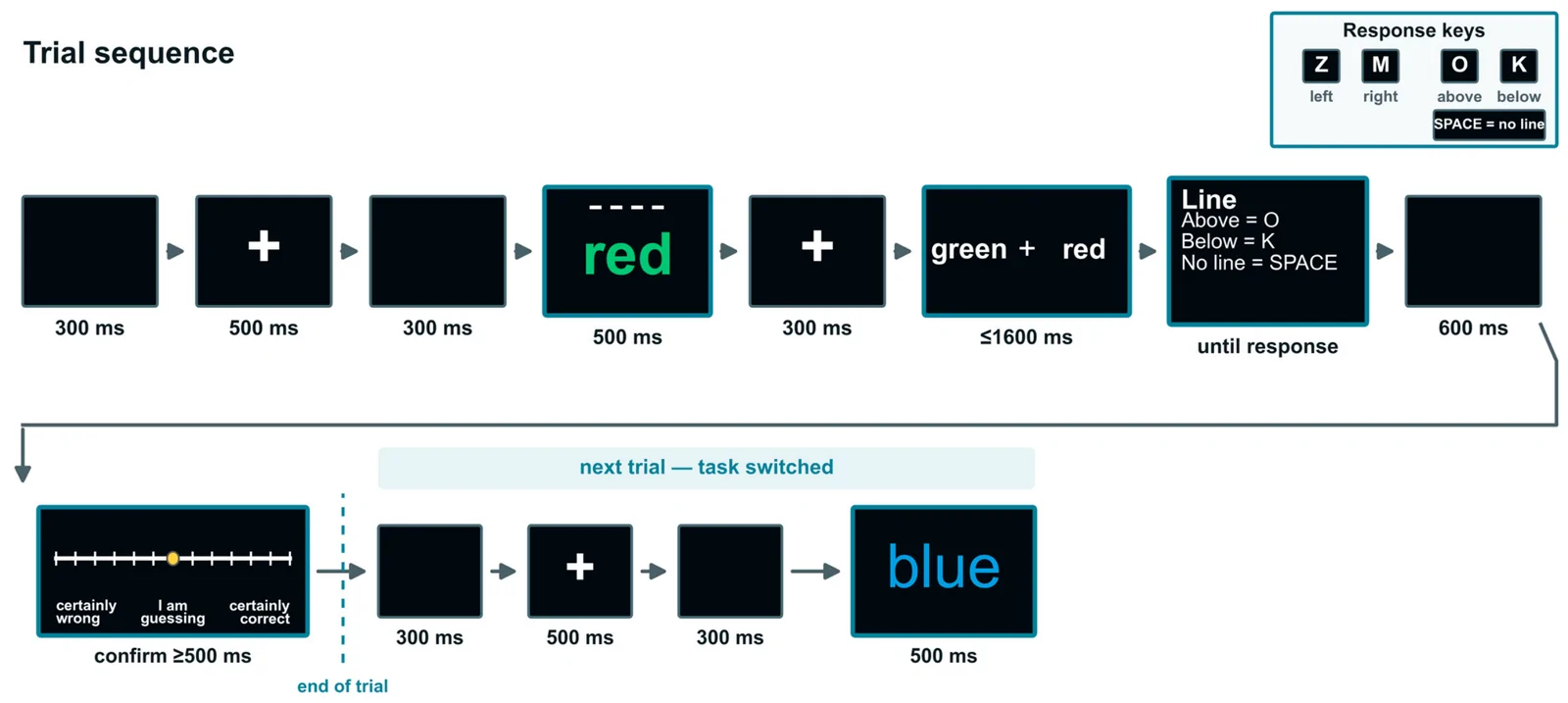
Experimental procedure in Experiment 1. Example sequence of two consecutive trials, showing target presentation, colour response, line-position judgment, and confidence rating. Arrows indicate the sequence of events within the trial, and the response-key inset shows the keys used for the colour and line-position judgments. In compound trials, a dashed line appeared above or below the colour word; the figure shows an example with the line above the word. The yellow marker indicates an example selected confidence rating. The vertical blue dashed line denotes the end of one trial and the transition to the next; the lower sequence illustrates a switch in stimulus complexity from a compound to a simple trial.

After the colour response, the word “Line” and a visual reminder of the line-response keys appeared on the screen. Participants then indicated whether the dashed line was absent, presented below the word, or presented above the word. They pressed the space bar with the right thumb to indicate no line, the “K” key to indicate a line below the word, and the “O” key to indicate a line above the word. This response was made without time pressure. After the line-position response, a black screen was presented for 600 ms. Participants then rated their confidence in the accuracy of their response on a 13-point scale. Importantly, participants evaluated their confidence in the entire response sequence, first identifying the colour and then the position of the line. The scale ranged from “certainly wrong” at point 1 to “certainly correct” at point 13. The midpoint, point 7, was labelled “I am guessing”; the remaining points were unlabelled. Participants moved along the scale using the “Z” and “M” keys and confirmed their selected rating with the space bar. To reduce accidental or impulsive confirmation, the selected point could be confirmed only after 500 ms. Confidence ratings were made without time pressure. After completing the experiment, participants were debriefed and paid for their participation. The entire procedure lasted approximately 60 min. The sequence of events within each experimental trial is illustrated in Figure 1.

### 2.2. Results

#### 2.2.1. Data Preparation

Accuracy was defined such that a trial was coded as correct only when both word colour and line-position responses were accurate. Accuracy was analyzed on all valid trials, whereas response times and confidence ratings were analyzed on correct trials only. For the response-time and confidence analyses, RTs more than 3 SD above the overall median (>1228.1 ms) or shorter than 100 ms were excluded. The first trial of the task and the first trial after each scheduled break were also excluded from these analyses. Higher confidence ratings indicated greater confidence that the response was correct, whereas lower ratings indicated greater confidence that an error had been made. We used an alpha level of 0.05 for all statistical tests. When applicable, post hoc comparisons were adjusted using the Tukey correction.

#### 2.2.2. Data Analysis

To examine whether conflict, complexity, and sequence affected performance and confidence in the modified Stroop task, we analyzed accuracy, response times, and confidence ratings as a function of three within-participant factors: conflict, complexity, and sequence. Across the mixed-effects analyses in both experiments, categorical predictors were coded using simple contrasts.

Accuracy was analyzed using a generalized mixed-effects logistic model with a binomial distribution and logit link function. The model included conflict, complexity, sequence, all two-way interactions, and the three-way interaction as fixed effects, with random intercepts for participants:

Accuracy ~ 1 + Conflict + Complexity + Sequence + Conflict × Complexity + Conflict × Sequence + Complexity × Sequence + Conflict × Complexity × Sequence + (1 | participant).

Response times and confidence ratings were analyzed using linear mixed-effects models with the same fixed-effects structure and random intercepts for participants. The response-time model was specified as follows:

RT ~ 1 + Conflict + Sequence + Complexity + Conflict × Sequence + Conflict × Complexity + Sequence × Complexity + Conflict × Sequence × Complexity + (1 | participant).

The confidence-rating model was specified as follows:

Confidence rating ~ 1 + Conflict + Sequence + Complexity + Conflict × Sequence + Conflict × Complexity + Sequence × Complexity + Conflict × Sequence × Complexity + (1 | participant).

Mixed-effects analyses were conducted in Jamovi (version 2.6.26) [45] using the GAMLj module (version 3.6.5) [46]. Models were estimated with the bobyqa optimizer and degrees of freedom for linear mixed-effects models were obtained using Satterthwaite’s approximation. All models achieved successful convergence. The inclusion of random intercepts allowed us to account for between-participant variability in baseline accuracy, response times, and confidence ratings.

For the exploratory signal-detection analyses, the colour decision provided a binary left- versus right-hand response structure, with the required response hand balanced equally across trials. Because confidence concerned the correctness of the complete colour-plus-line response sequence, first-order correctness was defined using the same criterion: a trial was considered correct only when both responses were correct. On all trials except those containing an isolated line-position error, the binary first-order response corresponded directly to the participant’s observed left- or right-hand colour response. When the colour response was correct but the subsequent line-position response was incorrect (3.5% of the primary SDT trials), the complete response sequence was nevertheless incorrect and was therefore represented as a first-order error. This approach aligned the first-order correctness criterion with the outcome evaluated by the confidence judgement while retaining the binary response structure required for the SDT analysis.

To assess the robustness of the SDT findings to the binary coding used in the primary analysis, we repeated the complete analysis after excluding all trials with an incorrect line-position response. This additional restriction removed 3.9% of the primary SDT trials. In the sensitivity analysis, performance on the additional response component was held constant as correct; first-order correctness was therefore determined entirely by the colour response, and the binary first-order response corresponded directly to the observed left- or right-hand choice on every included trial. All other preprocessing steps, confidence ratings, condition definitions, and estimation procedures were identical to those used in the primary analysis.

Confidence was entered on the original 13-point scale used in the task. First trials and trials with omitted responses were excluded before SDT estimation. First-order sensitivity (d′), metacognitive sensitivity (meta-d′), and metacognitive efficiency (M-ratio = meta-d′/d′) were estimated separately for the four Conflict × Complexity conditions using maximum-likelihood estimation in metadPy 0.1.2 [41,47,48]. Sequence was not included because further subdivision resulted in sparse participant-level cells. All 66 participants contributed estimates to both the primary and sensitivity analyses. The resulting estimates were analyzed using 2 × 2 repeated-measures ANOVAs with conflict and complexity as within-participant factors. Detailed model reports for all analyses in Experiments 1 and 2 are presented in the Supplementary Materials (Sections S1.1–S2.7).

#### 2.2.3. Accuracy

Firstly, we examined whether accuracy in the modified Stroop task was decreased by conflict, stimulus complexity, and sequence. Accuracy was analyzed using a generalized linear mixed-effects model with a binomial distribution and a logit link function. The model included conflict, complexity, sequence, and their interactions as fixed effects, with a random intercept for participant. The model was fitted to 29,502 trials from 66 participants. The fixed-effects structure was significant, χ^2^(7) = 426.56, *p* < 0.001. The marginal R^2^ was 0.08, whereas the conditional R^2^ was 0.23, suggesting additional between-participant variability in baseline accuracy beyond that captured by the fixed effects.

There were significant main effects of conflict, χ^2^(1) = 142.86, *p* < 0.001, complexity, χ^2^(1) = 212.45, *p* < 0.001, and sequence, χ^2^(1) = 48.73, *p* < 0.001. Overall, participants were more accurate in congruent than in conflict trials (M = 0.97 vs. 0.94, OR = 1.93, 95% CI [1.74, 2.15], *p* < 0.001), in simple than in compound trials (M = 0.97 vs. 0.94, OR = 2.23, 95% CI [2.01, 2.49], *p* < 0.001), and in repeated than in switched trials (M = 0.96 vs. 0.95, OR = 1.47, 95% CI [1.32, 1.64], *p* < 0.001). Accuracy was therefore affected by all three experimental manipulations.

There was a significant Conflict × Complexity interaction, χ^2^(1) = 8.48, *p* = 0.004. The conflict effect was significant for both simple and compound trials, but it was larger for simple trials (OR = 2.27, 95% CI [1.80, 2.87] vs. OR = 1.65, 95% CI [1.40, 1.93], respectively; *p* < 0.001 for both comparisons).

There was also a significant Complexity × Sequence interaction, χ^2^(1) = 39.83, *p* < 0.001. The sequence effect was significant for simple trials, whereas the corresponding comparison for compound trials did not reach significance. Participants were more accurate in simple-repeated than in simple-switched trials (M = 0.98 vs. 0.96, OR = 2.08, 95% CI [1.65, 2.63], *p* < 0.001). In contrast, the estimated difference between compound-repeated and compound-switched trials did not reach significance (both Ms = 0.94, OR = 1.04, 95% CI [0.88, 1.22], Tukey-adjusted *p* = 0.933). Thus, the sequence effect was larger for simple than compound trials. The Conflict × Sequence interaction, χ^2^(1) = 0.10, *p* = 0.753, and the three-way Conflict × Complexity × Sequence interaction, χ^2^(1) = 0.77, *p* = 0.381, did not reach significance.

**Figure 2:**
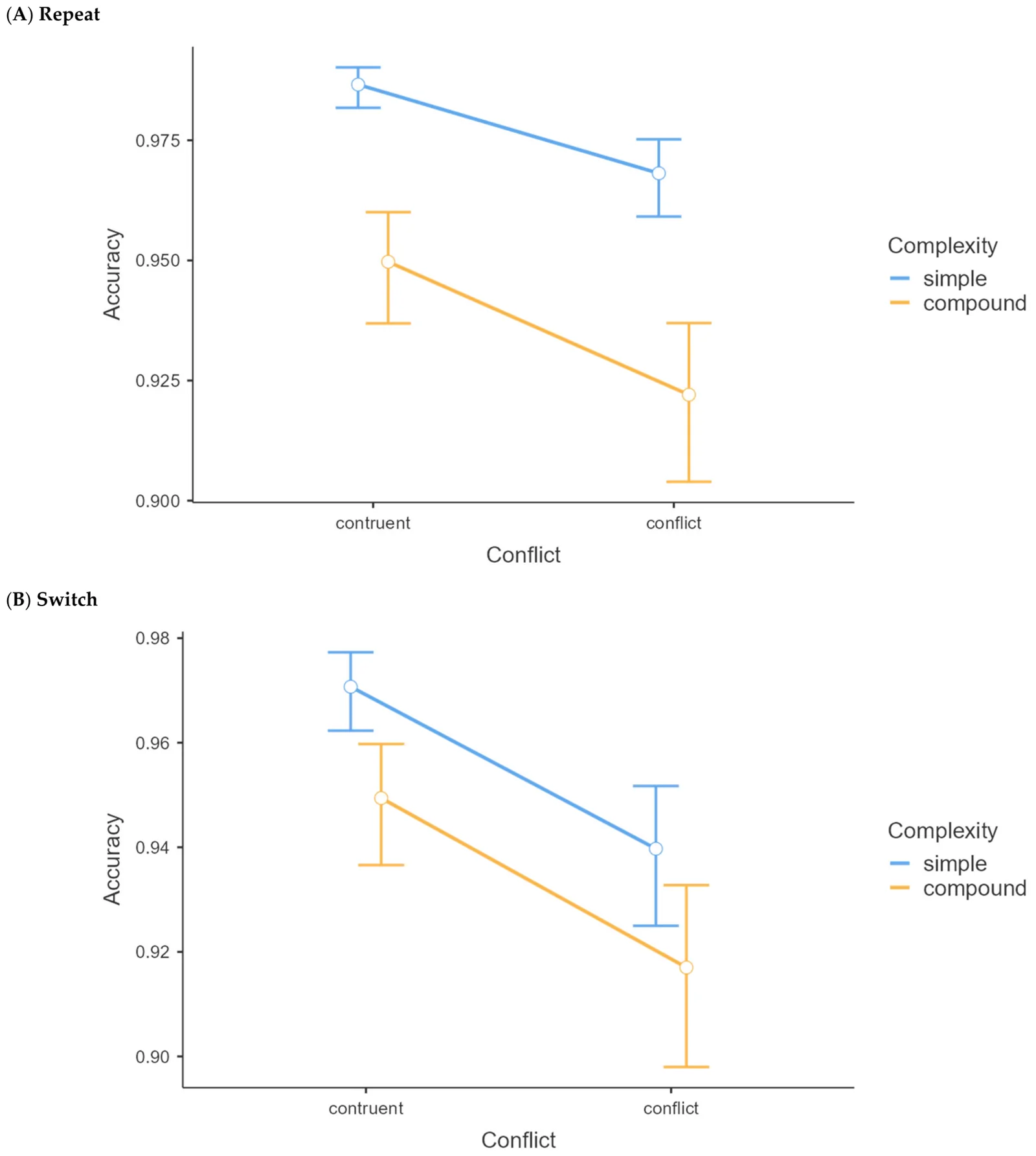
Estimated marginal means of response accuracy across experimental conditions. The upper panel represents repeat trials, whereas the lower panel represents switch trials in the modified Stroop task of Experiment 1.

In summary, participants were less accurate in conflict than in congruent trials, in compound than in simple trials, and in switched than in repeated trials. However, these effects were qualified by two interactions. First, the conflict effect was larger for simple than compound trials. Second, the sequence effect was larger for simple than compound trials: accuracy was lower in simple-switched than in simple-repeated trials, whereas the estimated difference between compound-switched and compound-repeated trials did not reach significance. The model-estimated accuracy pattern across conditions is presented in Table 1 and Figure 2.

#### 2.2.4. Response Times

Next, we examined whether response times in the modified Stroop task were modulated by conflict, stimulus complexity, and sequence. Response times were analyzed using a linear mixed-effects model with conflict, complexity, sequence, all two-way interactions, and the three-way interaction as fixed effects, with random intercepts for participants. The model was fitted to 26,787 observations from 66 participants. The fixed-effects structure was significant, χ^2^(7) = 1750.30, *p* < 0.001. Marginal and conditional R^2^ were 0.05 and 0.21, respectively.

There were significant main effects of conflict, F(1, 26,714.42) = 83.50, *p* < 0.001, and complexity, F(1, 26,715.31) = 1507.79, *p* < 0.001. Participants responded more slowly in conflict than in congruent trials (estimated difference = 20 ms, 95% CI [10, 20]) and in compound than in simple trials (estimated difference = 80 ms, 95% CI [70, 80]). The estimated overall difference between repeated and switched trials did not reach significance, F(1, 26,715.22) = 0.29, *p* = 0.592.

There were significant Conflict × Sequence, F(1, 26,714.85) = 88.52, *p* < 0.001, Conflict × Complexity, F(1, 26,714.24) = 42.10, *p* < 0.001, and Sequence × Complexity interactions, F(1, 26,714.28) = 54.26, *p* < 0.001. These two-way interactions were further qualified by a significant Conflict × Sequence × Complexity interaction, F(1, 26,715.07) = 24.05, *p* < 0.001. We therefore decomposed the three-way interaction by examining conflict and complexity effects separately in repeated and switched trials.

In repeated trials, the conflict effect depended on stimulus complexity. Conflict slowed responses for simple stimuli by 20 ms, *p* < 0.001, whereas for compound stimuli, the direction of the conflict effect was reversed: congruent–compound-repeated responses were 20 ms slower than conflict–compound-repeated responses, *p* < 0.001. The complexity effect was significant at both levels of conflict, but it was larger in congruent than conflict trials (110 vs. 70 ms; *p* < 0.001 for both comparisons).

In switched trials, conflict increased response times for both simple and compound stimuli by 40 and 30 ms, respectively (*p* < 0.001 for both comparisons). The complexity effect was also significant in switched trials and was estimated at 60 ms under both congruent and conflict conditions (*p* < 0.001 for both comparisons).

Finally, we examined the sequence effect within each Conflict × Complexity combination. In congruent–simple trials, the estimated difference between simple-switched and simple-repeated trials did not reach significance (Tukey-adjusted *p* = 0.660). In conflict–simple trials, participants responded more slowly in simple-switched than simple-repeated trials by 20 ms, *p* < 0.001. In congruent–compound trials, participants responded 40 ms faster in compound-switched than compound-repeated trials, *p* < 0.001. In conflict–compound trials, participants responded 10 ms more slowly in compound-switched than compound-repeated trials, Tukey-adjusted *p* = 0.006.

**Figure 3:**
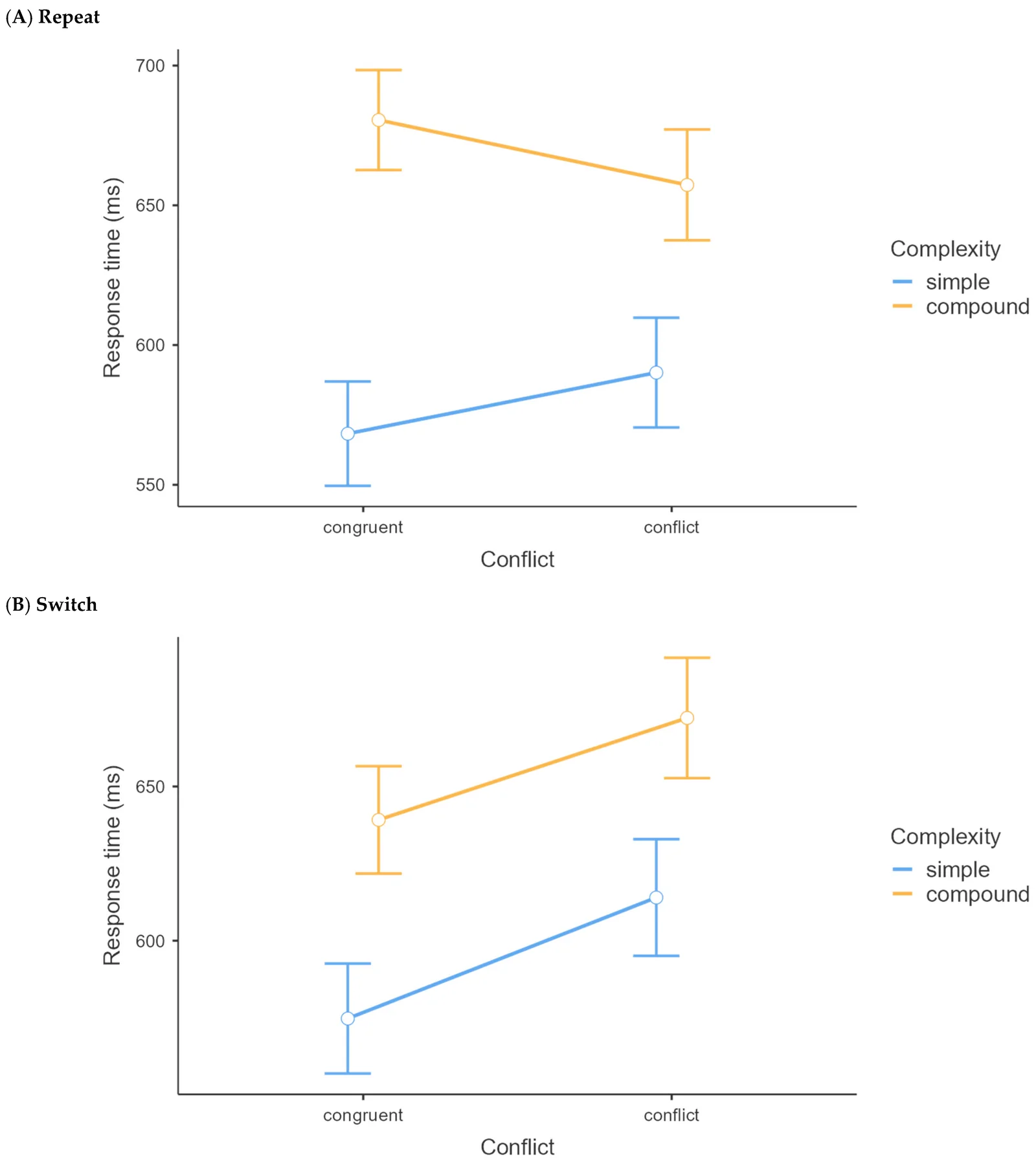
Estimated marginal means of response times across experimental conditions. The upper panel represents repeat trials, whereas the lower panel represents switch trials in the modified Stroop task in Experiment 1.

In summary, response times were longer in conflict than in congruent trials and in compound than in simple trials, whereas the estimated main effect of sequence did not reach significance. However, the significant three-way interaction showed that sequence effects depended jointly on conflict and stimulus complexity. In repeated trials, conflict slowed responses for simple stimuli but showed the opposite pattern for compound stimuli. In switched trials, conflict slowed responses for both simple and compound stimuli. Complexity increased response times in all conditions, but this effect was largest in congruent-repeated trials and smaller in conflict-repeated and switched trials. The condition-specific response-time pattern underlying the three-way interaction is shown in Table 2 and Figure 3.

#### 2.2.5. Confidence Judgments

Finally, we examined whether confidence ratings in the modified Stroop task were modulated by conflict, stimulus complexity, and sequence. Confidence ratings were analyzed using a linear mixed-effects model with conflict, complexity, sequence, all two-way interactions, and the three-way interaction as fixed effects, with random intercepts for participants. The model was fitted to 26,787 observations from 66 participants. The fixed-effects structure was significant, χ^2^(7) = 102.28, *p* < 0.001. Marginal and conditional R^2^ were 0.003 and 0.23, respectively.

There was a significant main effect of conflict, F(1, 26,714.21) = 19.85, *p* < 0.001. Participants reported lower confidence in conflict than in congruent trials (M = 12.50 vs. 12.56, b = −0.06, 95% CI [−0.09, −0.03]).

The main effects of sequence, F(1, 26,714.75) = 4.37, *p* = 0.037, and complexity, F(1, 26,714.81) = 11.65, *p* < 0.001, were also significant. When averaged across conditions, confidence was lower in switched than in repeated trials and in compound than in simple trials. Importantly, however, both marginal effects were qualified by the Sequence × Complexity interaction reported below.

There was a significant Sequence × Complexity interaction, F(1, 26,714.11) = 65.14, *p* < 0.001. The sequence effect was significant for both simple and compound trials, but it differed in direction and was larger for simple trials. Participants reported higher confidence in simple-repeated than simple-switched trials (M = 12.62 vs. 12.48, difference = 0.14, *p* < 0.001). For compound trials, the sequence effect was reversed: confidence was higher in compound-switched than compound-repeated trials (M = 12.54 vs. 12.46, difference = 0.08, *p* < 0.001). The Conflict × Sequence, Conflict × Complexity, and three-way interactions did not reach significance (all *p* values ≥ 0.755).

**Figure 4:**
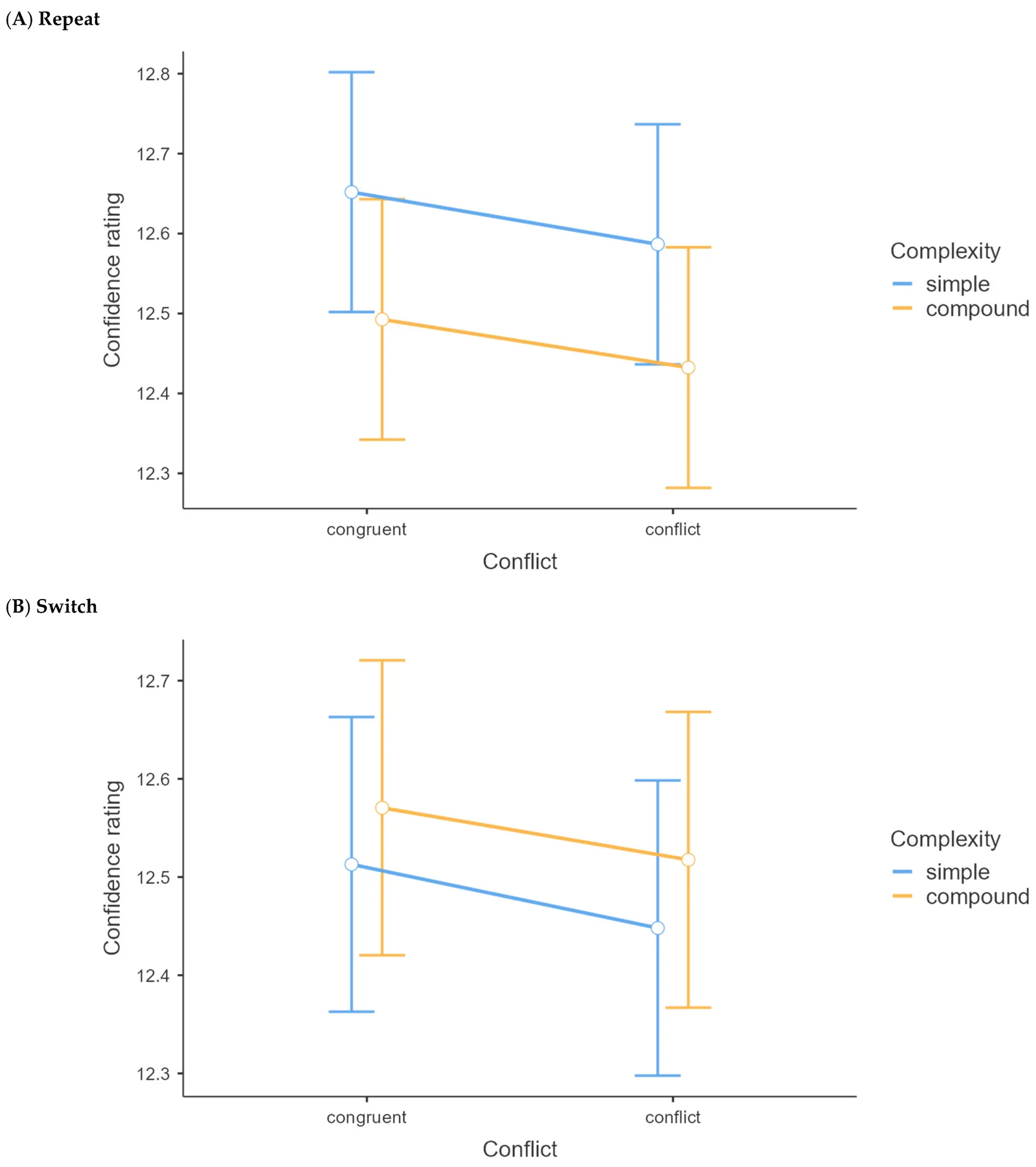
Mean confidence ratings across sequence, complexity, and conflict conditions in the modified Stroop task in Experiment 1. Error bars represent 95% confidence intervals. The bottom panel represents switched trials.

In summary, confidence was lower in conflict than in congruent trials, consistent with the view that conflict reduced subjective certainty, although this difference was small in absolute terms. Sequence and complexity also affected confidence, but their marginal effects were qualified by a crossover Sequence × Complexity interaction. Specifically, confidence was higher in simple-repeated than simple-switched trials, whereas the reverse pattern was observed for compound trials. The Sequence × Complexity interaction in confidence is shown in Table 3 and Figure 4.

#### 2.2.6. Exploratory Analyses of Metacognitive Sensitivity and Efficiency

We next examined first-order sensitivity and the sensitivity and efficiency of the corresponding confidence judgments across conflict and complexity conditions. First-order sensitivity was indexed by d′, metacognitive sensitivity by meta-d′, and metacognitive efficiency by M-ratio. Estimates were obtained separately for the four Conflict × Complexity conditions.

The analysis of d′ revealed significant main effects of conflict, F(1, 65) = 85.37, *p* < 0.001, ηp^2^ = 0.57, and complexity, F(1, 65) = 41.59, *p* < 0.001, ηp^2^ = 0.39. First-order sensitivity was lower in conflict than in congruent trials and in compound than in simple trials. The Conflict × Complexity interaction did not reach significance, F(1, 65) = 0.03, *p* = 0.868.

**Figure 5:**
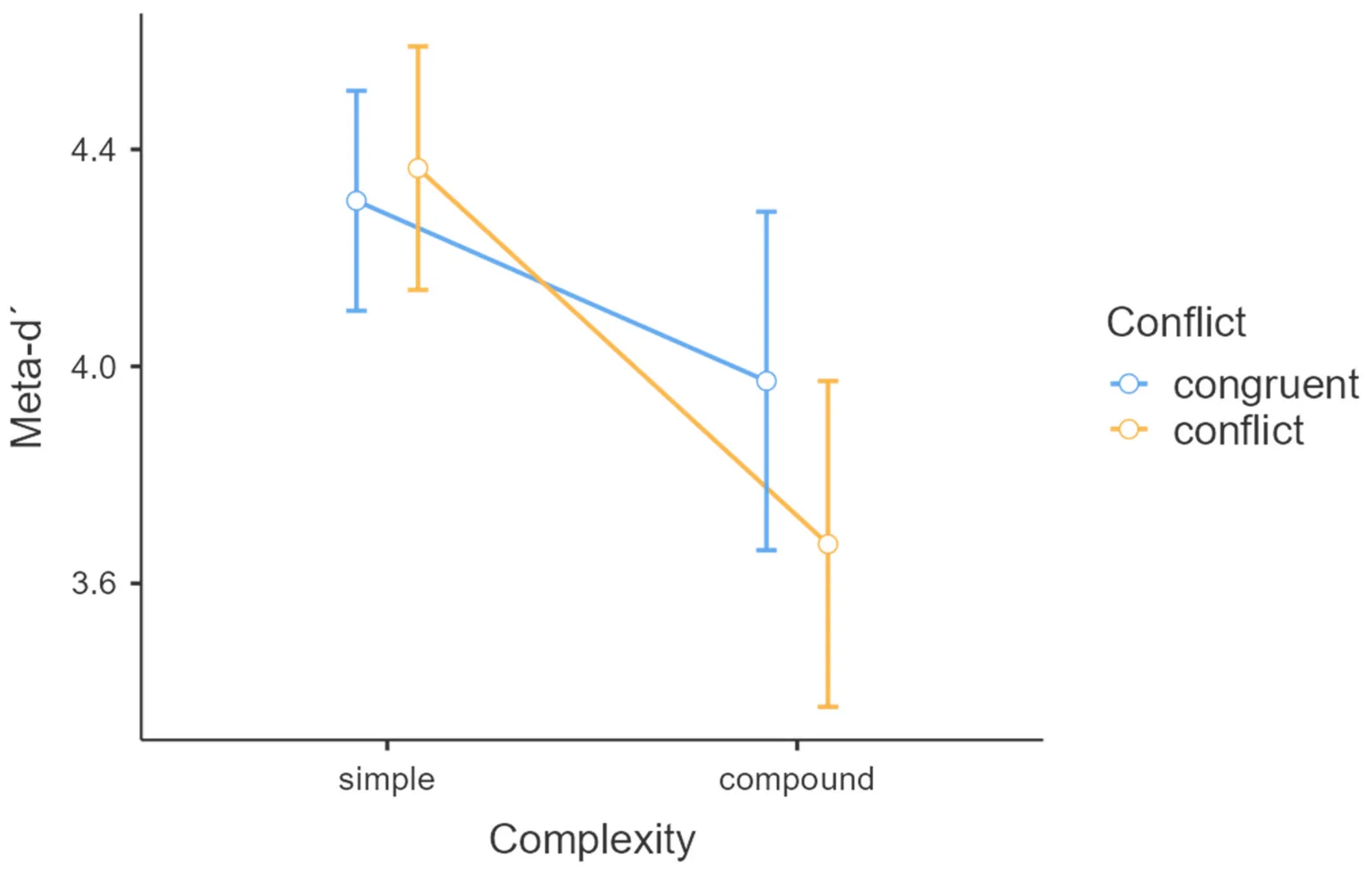
Metacognitive sensitivity—meta-*d′* in Experiment 1.

Meta-d′ showed a significant main effect of complexity, F(1, 65) = 22.71, *p* < 0.001, ηp^2^ = 0.26. Metacognitive sensitivity was higher in simple than compound trials. The main effect of conflict did not reach significance, F(1, 65) = 2.42, *p* = 0.125, ηp^2^ = 0.04. However, we observed a significant Complexity × Conflict interaction, F(1, 65) = 4.54, *p* = 0.037, ηp^2^ = 0.07. The interaction was most clearly reflected in the complexity effect: meta-d′ was substantially higher in simple than in compound trials under conflict (mean difference = 0.69, Tukey-adjusted *p* < 0.001), whereas the corresponding comparison under congruence did not reach significance after adjustment (mean difference = 0.33, Tukey-adjusted *p* = 0.083). The reduction in metacognitive sensitivity from simple to compound trials was larger under conflict than under congruence. The Conflict × Complexity pattern in meta-d′ is shown in Table 4 and Figure 5.

**Figure 6:**
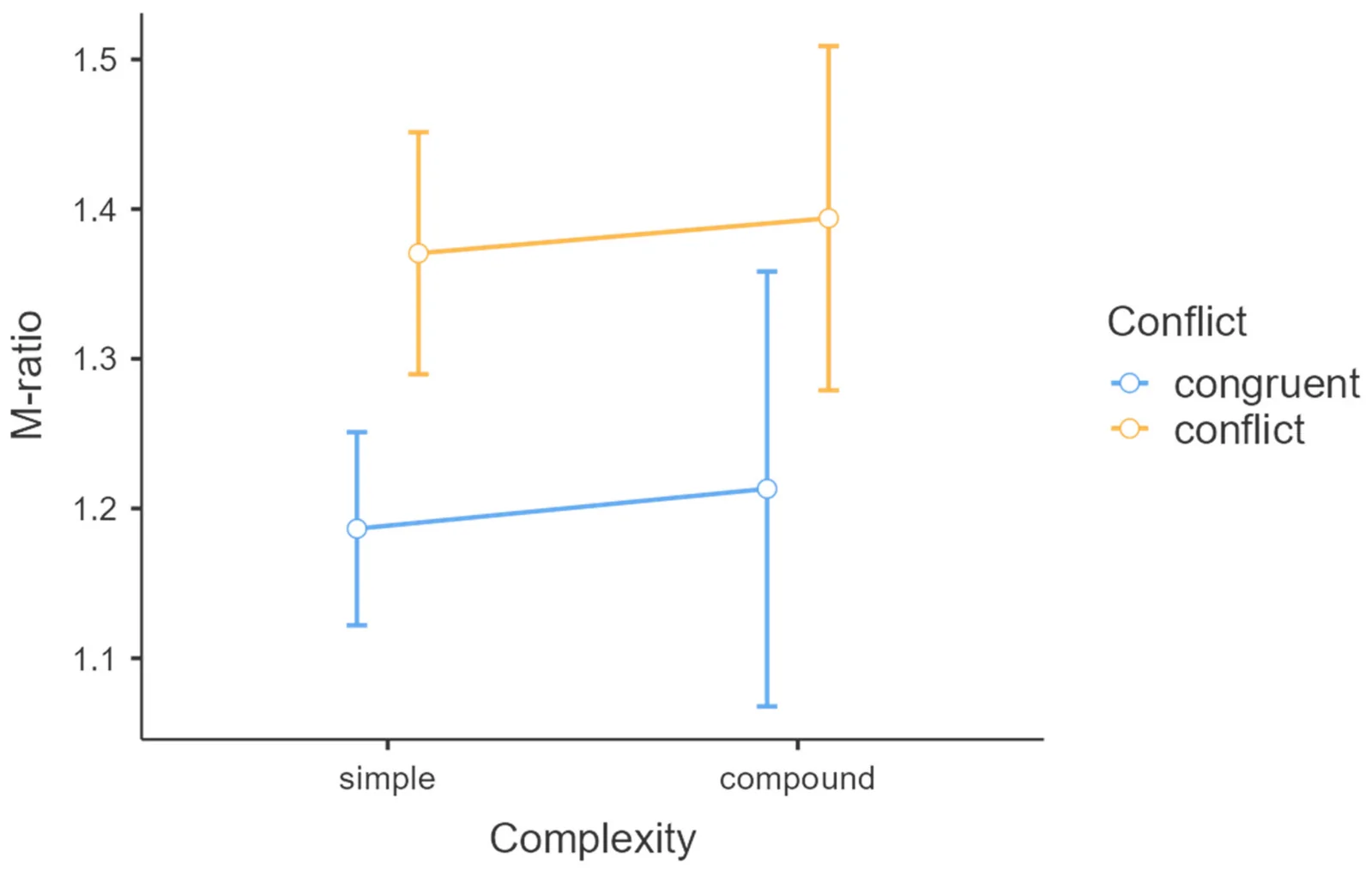
Metacognitive efficiency, M-ratio in Experiment 1.

We next quantified metacognitive efficiency with the M-ratio, which adjusts meta-d′ for primary-task sensitivity d′. Conflict had a significant main effect, F(1, 65) = 15.54, *p* < 0.001, ηp^2^ = 0.19: M-ratio was higher in conflict than in congruent trials. The effects of complexity, F(1, 65) = 0.28, *p* = 0.597, and Conflict × Complexity, F(1, 65) = 0.00, *p* = 0.971, did not reach significance. Because M-ratio expresses meta-d′ relative to d′, this result should not be interpreted as an absolute enhancement of metacognitive sensitivity. Table 5 and Figure 6 show the corresponding M-ratio estimates across conflict and complexity conditions.

To determine whether the SDT findings depended on the first-order coding used in the primary analysis, we repeated the analyses after excluding trials with an incorrect line-position response. Conflict continued to reduce d′, F(1, 65) = 91.68, *p* < 0.001, ηp^2^ = 0.59, whereas the complexity effect did not reach significance, F(1, 65) = 3.31, *p* = 0.073. Complexity continued to reduce meta-d′, F(1, 65) = 25.47, *p* < 0.001, ηp^2^ = 0.28, and the Conflict × Complexity interaction was again significant, F(1, 65) = 9.46, *p* = 0.003, ηp^2^ = 0.13. The simple–compound difference was significant under conflict (Tukey-adjusted *p* < 0.001), whereas the corresponding adjusted comparison under congruence did not reach significance (*p* = 0.052).

The conflict-related increase in M-ratio was also retained, F(1, 65) = 22.77, *p* < 0.001, ηp^2^ = 0.26, but was qualified by complexity: the Conflict × Complexity interaction was significant, F(1, 65) = 9.74, *p* = 0.003, ηp^2^ = 0.13. M-ratio was higher under conflict in simple trials (Tukey-adjusted *p* < 0.001), whereas the corresponding comparison in compound trials did not reach significance (Tukey-adjusted *p* = 0.383). M-ratio was also lower in compound than in simple trials under congruent and conflict conditions.

Taken together, excluding trials with incorrect line-position responses preserved the conflict-related reduction in *d′*, the complexity-related reduction in *meta-d′*, and the stronger expression of this effect under conflict, as well as the overall increase in *M-ratio* under conflict. However, the effect of complexity on *d′* was no longer significant. In addition, *M-ratio* exhibited both a main effect of complexity and a Conflict × Complexity interaction.

### 2.3. Interim Discussion of Experiment 1

In this experiment, we examined whether cognitive-control demands influence performance, reported confidence, and metacognitive monitoring in a modified Stroop task. We observed the effect of all three manipulations on response accuracy: it was lower in conflict compared to congruent trials, in compound compared to simple trials, and in switched compared to repeated trials. These findings suggest that experimental manipulations increased demands on the intended control processes: inhibition, updating, and shifting [38]. We assumed that the presence of conflicting stimuli would increase inhibition demands by requiring the cognitive system to resolve competition between the relevant stimulus dimension and the irrelevant word meaning [43]. Compound trials were assumed to increase updating demands by the requirement to maintain and process an additional line-position component, and switched trials required the cognitive system to shift between simple and compound stimuli.

We also observed the effect of conflict and complexity on response times. Responses were slower in conflict compared to congruent trials, and in compound compared to simple trials. Complexity increased response times across conditions, although the size of this effect varied depending on conflict and sequence. Thus, the response-time results confirmed the effects of conflict and stimulus complexity but also showed that the temporal pattern of performance depended on the specific combination of task conditions. These results closely resemble the pattern reported by Rietbergen et al. [39].

Surprisingly, in repeated trials, conflict increased response times to simple stimuli but reduced response times to complex stimuli. In switched trials, conflict slowed responses for both simple and compound stimuli. This pattern may reflect visual-working-memory dynamics. The complexity manipulation was intended to increase the need to update visual working memory by adding the line-position component to the colour-response requirement [38,39,40]. When a compound trial followed a simple trial, the line component may have been relatively salient because it introduced new task-relevant visual information. Novelty and visual salience can facilitate encoding into visual working memory [49,50]. By contrast, when compound trials appeared consecutively, participants had to update the current line-position representation in the context of recently processed line information from the preceding trial. This may have increased interference between adjacent visual representations, consistent with evidence that previously stored items can interfere with current visual-working-memory contents [51,52]. Thus, switching between simple and compound trials may have affected memory-demanding trials differently than trials requiring conflict resolution. The novelty effect may have been more pronounced in congruent trials, where word meaning did not activate a competing response [43,53]. Participants may therefore have been more able to postpone the colour response and accumulate information about line position. This could explain why congruent compound-repeated trials produced the longest RTs.

The main question of Experiment 1 was whether increased cognitive-control demands influenced confidence in response correctness. The confidence analysis was restricted to objectively correct trials. Participants reported lower confidence in conflict than in congruent trials even when the final task response was correct. The conflict-related difference was statistically reliable but small in absolute magnitude (0.06 points on the 13-point scale), and the Conflict × Complexity, Conflict × Sequence, and three-way interactions did not reach significance. Confidence was also affected by complexity and sequence, but these effects were qualified by a crossover interaction. Confidence was higher in simple-repeated than in simple-switched trials, whereas confidence was higher in compound-switched than in compound-repeated trials. Thus, there was no uniform confidence cost of switching. Instead, confidence depended on the relation between the complexity of the current trial and that of the preceding trial. Because this interaction was not predicted, its interpretation should remain cautious.

The exploratory SDT analyses showed that conflict and complexity affected first-order and metacognitive performance differently. Conflict substantially reduced first-order sensitivity but did not produce an overall reduction in meta-*d′*. Correspondingly, M-ratio was higher under conflict than under congruent conditions. Because M-ratio expresses meta-*d′* relative to *d′*, this pattern indicates that first-order sensitivity declined more strongly than metacognitive sensitivity under conflict; it should not be interpreted as an absolute improvement in metacognitive monitoring [41,48]. Complexity showed a different pattern. Meta-*d′* was lower in compound than in simple trials, and this reduction was strongest when compound processing occurred under conflict. Importantly, this effect was also observed when trials with an incorrect line-position response were excluded, whereas the corresponding complexity effect on *d′* was no longer detected. The sensitivity analysis therefore strengthens the conclusion that the complexity-related reduction in metacognitive sensitivity does not depend on the particular treatment of trials containing line-position errors in the primary SDT representation. Together, these findings suggest that increased stimulus complexity compromises metacognitive sensitivity, particularly when additional processing demands coincide with response conflict. This pattern is consistent with evidence that active manipulation of working-memory contents can impair metacognitive monitoring [35].

## 3. Experiment 2

In Experiment 1, we tested the effect of increased task demands on confidence in one’s response in a perceptual task. In this experiment, we measured confidence in the occurrence of a partial error during responding to the same task. The goal was to find out how the assessment of the response process itself is affected by conditions requiring inhibition, updating, and switching.

We used electromyography (EMG) to detect covert motor activations that are precisely located in time but remain invisible at the level of overt behaviour [11,12,13]. This approach makes it possible to distinguish confidence in final response correctness from confidence in response execution, because an overtly correct response may still include a covert incorrect motor activation [11,12,13]. Participants often remain unaware of such partial errors, despite their efficient correction [11,12,13]. However, previous studies have shown that people can detect partial errors on the basis of motor-process characteristics, such as the strength of the incorrect motor activation and the time needed to correct it [12,13,25]. Building on previous EMG research, we examined whether confidence that a partial error had occurred was associated with objectively measured characteristics of the partial-error event and whether these associations varied across cognitive-control conditions. We focused on two EMG-derived characteristics of the event: incorrect-burst surface and correction time. Previous studies have reported associations between partial-error magnitude, correction dynamics, and both partial-error detection and confidence [12,13,25]. Accordingly, we expected greater incorrect-burst surface and longer correction time to be associated with higher partial-error confidence. Critically, we tested whether these associations differed across conflict, complexity, and sequence conditions.

Additionally, to explore the temporal dynamics of cognitive control, we decomposed response times into premotor and motor components using EMG. Following the classical fractionated reaction-time approach, premotor time was defined as the interval between stimulus onset and response-related EMG onset, whereas motor time was defined as the interval between EMG onset and the overt mechanical response [54,55]. This decomposition distinguished the interval preceding measurable muscle activation from the interval between EMG onset and overt response execution.

We additionally analyzed the objective occurrence of partial errors among fully correct trials. This analysis was necessary to distinguish changes in confidence from changes in the actual frequency of covert incorrect motor activations. Higher partial-error confidence in a particular condition could reflect a greater number of partial errors, an expectation that partial errors were more likely, or a change in the correspondence between EMG-derived response-process characteristics and confidence.

Finally, we used exploratory signal-detection analyses to characterize initial-motor-response sensitivity together with the metacognitive sensitivity and efficiency of partial-error judgments. Initial-motor-response sensitivity was defined by the correspondence between the response hand required by the colour stimulus and the hand in which response-related EMG activity first emerged. Accordingly, d′ indexed sensitivity to this stimulus-response correspondence, whereas meta-d′ and M-ratio characterized metacognitive sensitivity and efficiency with respect to the same representation.

### 3.1. Materials and Methods

Experiment 2 used a modified Stroop task closely matched to Experiment 1 but adapted for EMG recording and partial-error confidence judgments. The aim was to examine whether participants could monitor partial errors, defined as covert incorrect motor activations that were corrected before producing an overt error. EMG activity was recorded from both hands during the colour response, allowing us to identify trials in which an incorrect response was partially activated but successfully corrected.

#### 3.1.1. Participants

Forty-nine volunteers were enrolled in the study (29 women and 20 men; age range: 18–35 years, *M* = 25.3, *SD* = 4.5). Data were collected between October and November 2023. Two participants completed technical pilot sessions used to verify electrode placement, EMG acquisition settings, and the instructions for the partial-error confidence scale. Minor technical and instructional adjustments were introduced after these sessions. Because the final experimental and recording settings had not yet been established, the pilot data were not included in the main analyses. The final analytical sample therefore comprised 47 participants. All participants were native Polish speakers, reported no history of neurological disorders, had normal or corrected-to-normal vision, and reported normal colour vision. All participants provided written informed consent before the study. The study was approved by the Research Ethics Committee of the Institute of Psychology, Jagiellonian University (opinion no. KE/5_2023; 16 February 2023). Participants received approximately 10.36 EUR for their participation.

#### 3.1.2. Materials

The task was adapted from the modified Stroop task used in Experiment 1. Participants first responded to the font colour of a colour word while ignoring its meaning and then reported the presence and position of an additional line component. This design allowed us to manipulate inhibitory control, stimulus complexity, and sequence-related switching demands within the same task.

Each stimulus was one of four Polish colour words: “czerwony” [red], “niebieski” [blue], “różowy” [pink], and “zielony” [green]. Each word was displayed in one of four font colours: red, blue, pink, or green. Red and pink were assigned to the left-hand response, whereas green and blue were assigned to the right-hand response. Responses were collected with a Cedrus RB-830 response box (Cedrus, San Pedro, CA, USA).

As in Experiment 1, the task manipulated three factors: conflict, complexity, and sequence. Conflict referred to whether word meaning and font colour were congruent or incongruent. Complexity referred to whether the stimulus was simple or compound. Simple stimuli consisted only of the colour word, whereas compound stimuli included the colour word and an additional dashed line positioned above or below it. Sequence referred to whether stimulus complexity repeated or changed from the preceding trial, yielding simple-repeated, simple-switched, compound-repeated, and compound-switched trials.

To minimize low-level learning and stimulus repetition, two alternating stimulus lists were used. The words “niebieski” and “zielony” appeared on odd trials, whereas “czerwony” and “różowy” appeared on even trials. The task consisted of 512 trials: 128 simple-congruent trials, 128 compound-congruent trials, 128 simple-conflict trials, and 128 compound-conflict trials. One participant completed 600 trials because the experimental task was restarted following a technical issue with the recording apparatus at the beginning of the session.

#### 3.1.3. Partial-Error Instruction and Confidence Scale

Before the experimental task, participants received a detailed explanation of partial errors. They were informed that, on some trials, they might begin to activate the hand associated with the incorrect response but interrupt this activation before pressing the incorrect button and subsequently producing the correct response. A partial error was therefore described as a covert incorrect motor activation that was corrected before it resulted in an overt error.

To make the concept directly observable, participants were shown their own live EMG signals from both hands on a monitor. They were asked to press the response buttons and observe how the corresponding muscle activity changed in real time. They were also shown visual examples of pure-correct responses, partial errors, and overt errors and received detailed instructions on how these events should be reported using the confidence scale. This procedure followed previous partial-error studies in which participants were familiarized with partial errors using example traces and their own ongoing EMG activity [12].

The original Polish response labels are reported here in English translation. The scale consisted of seven options: 1 = “certainly not a partial error,” 2 = “probably not a partial error,” 3 = “maybe not a partial error,” 4 = “maybe a partial error,” 5 = “probably a partial error,” 6 = “certainly a partial error,” and 7 = “error.” Ratings from 1 to 6 therefore represented graded confidence concerning the absence or presence of a partial error, with higher values indicating greater confidence that a partial error had occurred, whereas rating 7 indicated that the participant believed that an overt error had occurred. Before the main experimental blocks, participants were again shown examples of partial errors and reminded how to use the scale.

#### 3.1.4. Procedure

The procedure was run using PsychoPy software (version 2023.1) [44] in the Laboratory of the Department of Experimental Psychology at the Institute of Psychology, Jagiellonian University. After providing written informed consent, participants were prepared for EMG recording and seated in front of the monitor with both hands placed on the response box. The experiment began with two training sessions. The first training session consisted of 24 trials and was designed to help participants learn the colour-to-response mappings. The second training session consisted of 32 trials and used the same structure as the main task, but without the partial-error confidence scale. Before the main task, participants again saw visual examples of partial errors and received a reminder of the confidence-scale instructions. Each experimental trial began with a blank screen for 300 ms, followed by a fixation point for 500 ms and another blank screen for 300 ms. The target colour-word stimulus was then presented for 500 ms. Participants responded to the font colour using the left or right response button and had up to 1600 ms to respond. If no response was made within this window, the trial ended with the feedback “Too slow.” After the colour response, participants reported the position of the line component on a three-point scale: “down,” “absent,” and “up.” They moved the indicator using the left and right thumbs and confirmed the selected position with the right response button. To prevent impulsive confirmation, the response could be confirmed only after 230 ms. The line-position response was not time-limited. Finally, at the end of each trial, participants rated their confidence that a partial error had occurred during the colour response. The selected confidence rating could also be confirmed only after 230 ms. Confidence ratings were not time-limited. The confidence judgement was therefore retrospective and concerned whether a partial error had occurred during the preceding colour response.

#### 3.1.5. EMG Recording and Processing

Surface EMG provides a time-resolved measure of electrical activity associated with recruitment of the recorded muscle. Here, activity of the flexor pollicis brevis provided a peripheral marker of thumb-related response activation. Bipolar surface EMG activity was recorded using a BioSemi ActiveTwo system (BioSemi, Amsterdam, The Netherlands). For each hand, two flat active Ag/AgCl electrodes were attached approximately 2 cm apart over the thenar eminence. The skin over the electrode sites was cleaned before electrode placement. Signals were sampled at 2048 Hz. The BioSemi CMS/DRL common-mode feedback system was used instead of a conventional recording reference and ground. Offline, right- and left-hand bipolar EMG channels were obtained by subtracting EXG2 from EXG1 and EXG4 from EXG3, respectively.

Before the task, the quality of the bilateral EMG recordings was checked while participants voluntarily pressed the response buttons and viewed their own live muscle activity. During the experiment, both EMG traces were continuously monitored by the experimenter. When tonic background activity or recording noise was visible and could obscure small task-related activations, the participant was asked to relax the hand muscles before continuing. This procedure followed established partial-error recording protocols [12,13].

EMG preprocessing and initial burst detection were performed in Python 3.13.5 using MNE 1.9.0 and the DEBUT 0.0.1 workflow developed by Laure Spieser and Boris Burle. The two bipolar EMG channels were high-pass-filtered offline at 10 Hz. EMG-burst onsets and offsets were initially identified automatically using the DEBUT onset-detection procedure.

All automatically generated onset and offset markers were subsequently inspected visually. Marker correction was performed before trial classification by a researcher blind to the experimental condition and to the participant’s partial-error confidence rating. False-positive detections were removed, missed bursts were added, and onset or offset markers were repositioned when they did not correspond to the visually identifiable beginning or end of phasic EMG activity. This blinded visual verification was used to minimize the possibility that knowledge of the task condition or subjective report influenced EMG classification, consistent with previous partial-error protocols [12,13].

Trials were classified using the overt response and the verified temporal pattern of bilateral EMG activity. Pure-correct trials were behaviourally correct trials containing a response-related EMG burst in the correct hand without a preceding incorrect-hand burst. Partial-error trials were behaviourally correct trials in which a subthreshold activation in the incorrect hand preceded the subsequent correct-hand activation. Overt-error trials were trials on which the incorrect mechanical response was produced.

A trial was excluded from EMG-based analyses when tonic background activity or a transient artefact prevented reliable identification of a burst; when an onset or offset could not be determined unambiguously; when overlapping, multiple, or otherwise irregular activations prevented reliable classification; or when the necessary stimulus, response, or EMG markers were absent. Trials with an incorrect colour response or an incorrect line-position response were additionally excluded from analyses restricted to fully correct trials.

Premotor time was defined as the interval between stimulus onset and the onset of the correct-hand EMG burst. Motor time was defined as the interval between correct-hand EMG onset and the overt button press. Correction time was defined as the interval between the onset of the incorrect-hand burst and the onset of the subsequent correct-hand burst.

Incorrect-burst surface was calculated from the rectified EMG activity between the manually verified onset and offset of the incorrect-hand activation. For each partial-error trial, a custom Python script selected the channel associated with the incorrect response and summed the absolute signal amplitudes between these two markers. Larger surface values therefore reflected a stronger, longer-lasting, or both stronger and longer-lasting incorrect motor activation.

**Figure 7:**
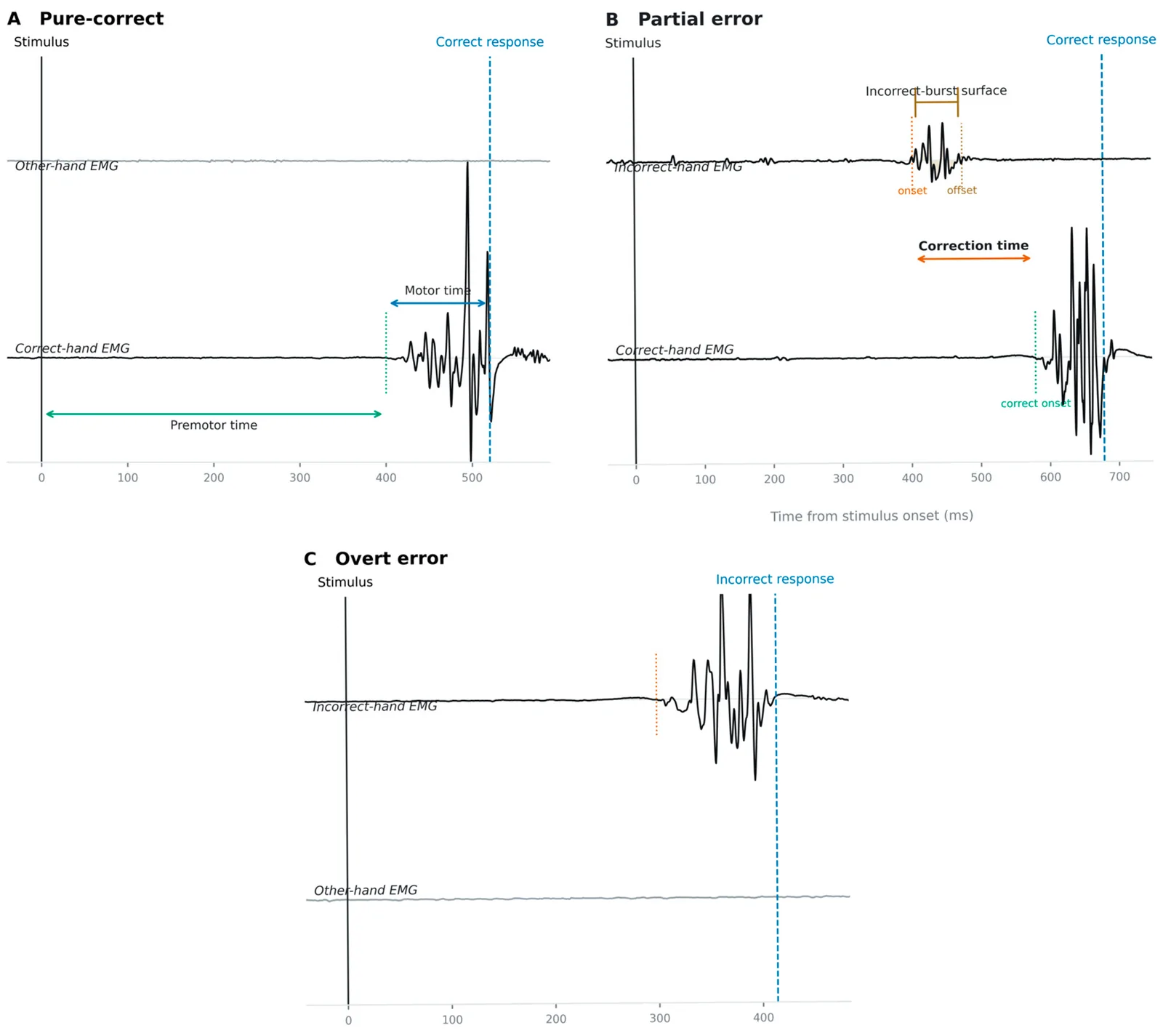
Schematic illustration of the three principal response patterns in Experiment 2. Orange and green dashed lines indicate the onset of EMG activity in the incorrect-response and correct-response hand, respectively. Blue dashed lines indicate the time at which the response was registered by the computer. (**A**) Pure-correct trial: response-related EMG activity emerges in the correct hand and culminates in a correct button press, with no preceding incorrect-hand activation. Premotor time was defined as the interval between stimulus onset and correct-hand EMG onset, and motor time as the interval between correct-hand EMG onset and the overt response. (**B**) Partial-error trial: a subthreshold incorrect-hand EMG burst is followed by correct-hand activation and a correct button press. Correction time was defined as the interval between incorrect-hand EMG onset and the onset of the subsequent correct-hand EMG burst. The incorrect-burst surface was quantified between the manually verified onset and offset of the incorrect-hand EMG burst. (**C**) Overt-error trial: response-related EMG activity in the incorrect hand culminates in an incorrect button press.

The three principal response patterns used in Experiment 2, together with the EMG-derived measures used in subsequent analyses, are illustrated schematically in Figure 7.

#### 3.1.6. Signal-Detection and Metacognitive Analyses

For the exploratory signal-detection analyses, only fully correct trials classified by EMG as either pure-correct or partial-error trials were included. The binary stimulus class was defined by the response hand required by the colour stimulus (left vs. right), whereas the binary response class was defined by the hand in which the first response-related EMG burst was detected (left vs. right). In pure-correct trials, the first EMG activation occurred in the stimulus-required hand, whereas in partial-error trials, it occurred in the opposite hand and was subsequently corrected before the overt response. Thus, although both trial types culminated in a correct overt response, they differed with respect to the hand of the initial EMG-defined motor response. The SDT representation was therefore based on the correspondence between the task-defined stimulus class and the hand of the initial EMG-defined response. Accordingly, d′ indexed sensitivity to this stimulus–response correspondence rather than overt behavioural accuracy.

For the SDT analysis, partial-error ratings from 1 to 6 were retained as six ordered confidence levels. Because higher original ratings indicated greater confidence that a partial error had occurred, ratings were reverse-coded before meta-d′ estimation (Confidence = 7 − original rating), so that higher values indicated greater confidence that no partial error had occurred. Rating 7 indicated an overt error and was not included.

Metacognitive sensitivity was quantified as meta-d′ and metacognitive efficiency as M-ratio (meta-d′/d′) [41,48]. Meta-d′ indexed how effectively participants’ graded confidence judgments discriminated between trials in which the initial EMG-defined response occurred in the stimulus-required hand (pure-correct trials) and trials in which it occurred in the opposite hand (partial-error trials). Estimates were obtained separately for the four Conflict × Complexity conditions using maximum-likelihood estimation in metadPy 0.1.2 [47] with six confidence levels. After restricting the SDT input to fully correct pure-correct and partial-error trials with ratings from 1 to 6 and valid response-hand codes, 15,494 trials from 47 participants were available for participant-level estimation. Sequence was not included because further subdivision resulted in sparse participant-level cells.

Of the 47 participants contributing eligible trials, one had no eligible observations in one Conflict × Complexity condition, and three had insufficient data in at least one condition to obtain participant-level estimates. One additional participant was excluded because their d′ estimates fell more than three sample standard deviations below the corresponding condition median in all four Conflict × Complexity conditions. The repeated-measures analyses of d′, meta-d′, and M-ratio therefore included 42 participants.

### 3.2. Results

#### 3.2.1. The Effect of Conflict, Complexity, and Sequence on Overt Response Accuracy

Firstly, we examined the influence of conflict, stimulus complexity, and sequence on response accuracy in the Stroop test. For this purpose, we fitted a generalized linear mixed-effects model with a binomial distribution and a logit link function. The model included conflict, complexity, sequence, and their interactions as fixed effects and a random intercept for participant. The model was fitted to 21,049 trials from 47 participants. The fixed-effects structure was significant, χ^2^(7) = 174.82, *p* < 0.001. The marginal R^2^ was 0.02, whereas the conditional R^2^ was 0.24.

There was a significant main effect of conflict, χ^2^(1) = 163.91, *p* < 0.001. Participants responded more accurately in congruent than in conflict trials (M = 0.89 vs. 0.83, OR = 1.66, 95% CI [1.53, 1.79]). Thus, conflict between task-relevant and task-irrelevant information reduced overt response accuracy. The main effects of complexity, χ^2^(1) = 0.00, *p* = 0.969, and sequence, χ^2^(1) = 0.81, *p* = 0.367, did not reach significance.

The Complexity × Sequence interaction was significant, χ^2^(1) = 3.96, *p* = 0.047. However, this two-way interaction was qualified by a significant Conflict × Complexity × Sequence interaction, χ^2^(1) = 5.40, *p* = 0.020. We therefore decomposed the three-way interaction by examining the conflict effect separately within each combination of complexity and sequence.

The conflict effect was significant in all four Complexity × Sequence conditions, but its magnitude varied across conditions. In repeated trials, the conflict effect was significant for simple and compound stimuli (OR = 1.79 and 1.58, respectively; *p* < 0.001 for both comparisons), and the difference in its magnitude did not reach significance, χ^2^(1) = 1.23, *p* = 0.267. In switched trials, the conflict effect was again significant for both levels of complexity (compound: OR = 1.85; simple: OR = 1.45; *p* < 0.001 for both comparisons), but it was larger in compound-switched than simple-switched trials, χ^2^(1) = 4.70, *p* = 0.030.

**Figure 8:**
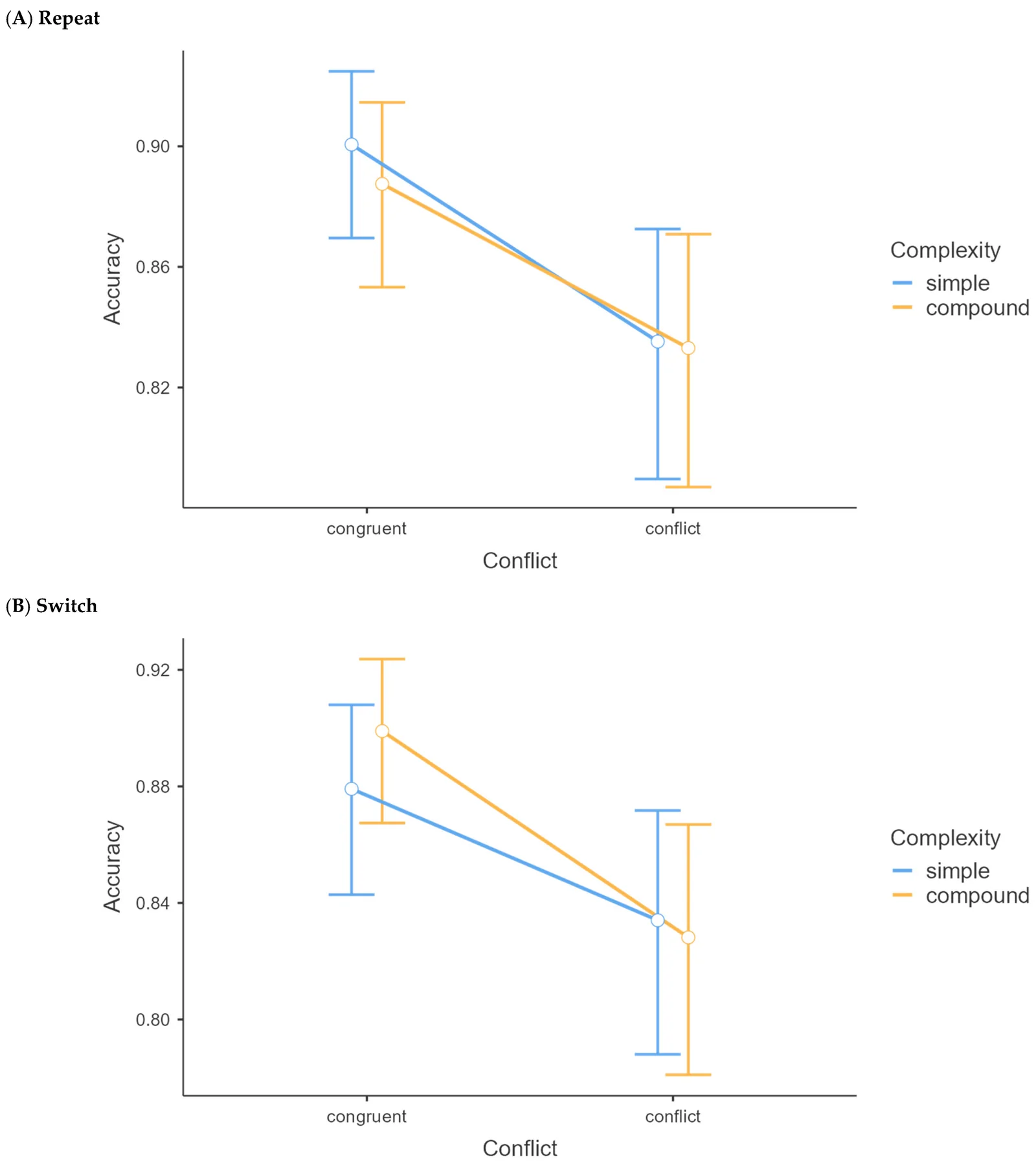
Model-estimated response accuracy across task conditions in Experiment 2. Estimated marginal means are expressed as probabilities. Error bars represent 95% confidence intervals.

In summary, response accuracy was lower in conflict than in congruent trials, reflecting a robust interference cost in Experiment 2. The main effects of complexity and sequence did not reach significance. However, the significant three-way interaction showed that the magnitude of the conflict effect depended on the combination of complexity and sequence. The conflict effect was detected in all conditions; its magnitude differed between complexity conditions in switched trials, but not reliably in repeated trials. The pattern of conflict effects across Complexity × Sequence conditions is shown in Table 6 and Figure 8.

#### 3.2.2. Partial-Error Occurrence

We next examined whether the experimental conditions affected partial-error occurrence in fully correct trials. The analysis was restricted to eligible trials in which both the colour response and the subsequent line-position response were correct and for which a valid EMG classification was available. Within this set, partial-error occurrence was coded as present when a verified incorrect-hand activation preceded the subsequent correct-hand activation and as absent otherwise. A binomial generalized linear mixed-effects model included conflict, complexity, sequence, and their interactions as fixed effects and a random intercept for participant. The model was fitted to 17,413 trials from 47 participants. The fixed-effects structure was significant, χ^2^(7) = 25.52, *p* < 0.001; marginal R^2^ was 0.004 and conditional R^2^ was 0.14. A main effect of conflict was detected, χ^2^(1) = 10.92, *p* = 0.001. Among trials in which both task responses were correct, partial errors were more likely in conflict than in congruent trials (M = 0.108 vs. 0.093, OR = 1.18). A main effect of complexity was also detected, χ^2^(1) = 11.81, *p* < 0.001. Partial errors were less likely in compound than in simple trials (M = 0.093 vs. 0.109, OR = 0.84). The sequence effect did not reach significance, χ^2^(1) = 0.49, *p* = 0.482, and no interaction term reached significance (all *p* values ≥ 0.358). Thus, among trials in which both the colour and line-position responses were correct, partial errors occurred more frequently under conflict and less frequently in compound than in simple trials.

#### 3.2.3. The Effect of Conflict, Complexity, and Sequence on Response Times

We then examined whether conflict, stimulus complexity, and sequence affected the latency of correct overt responses. Response times were analyzed to determine whether Experiment 2 reproduced the costs observed in Experiment 1 and previous work [39]. A linear mixed-effects model with conflict, complexity, sequence, and their interactions as fixed effects and a random intercept for participants was estimated on 17,413 fully correct trials from 47 participants. The fixed-effects structure was significant, χ^2^(7) = 375.04, *p* < 0.001; marginal R^2^ was 0.01 and conditional R^2^ was 0.46.

There were significant main effects of conflict, F(1, 17,361) = 286.02, *p* < 0.001, and complexity, F(1, 17,359) = 78.20, *p* < 0.001. Response times were longer in conflict than in congruent trials (estimated interference cost = 37.95 ms, 95% CI [33.56, 42.34]) and in compound than in simple trials (estimated complexity cost = 19.71 ms, 95% CI [15.34, 24.08]). The estimated sequence effect was small and did not reach significance, F(1, 17,359) = 3.61, *p* = 0.057 (b = 4.24 ms, 95% CI [−0.13, 8.61]). No interaction term reached significance (all *p* values ≥ 0.065).

**Figure 9:**
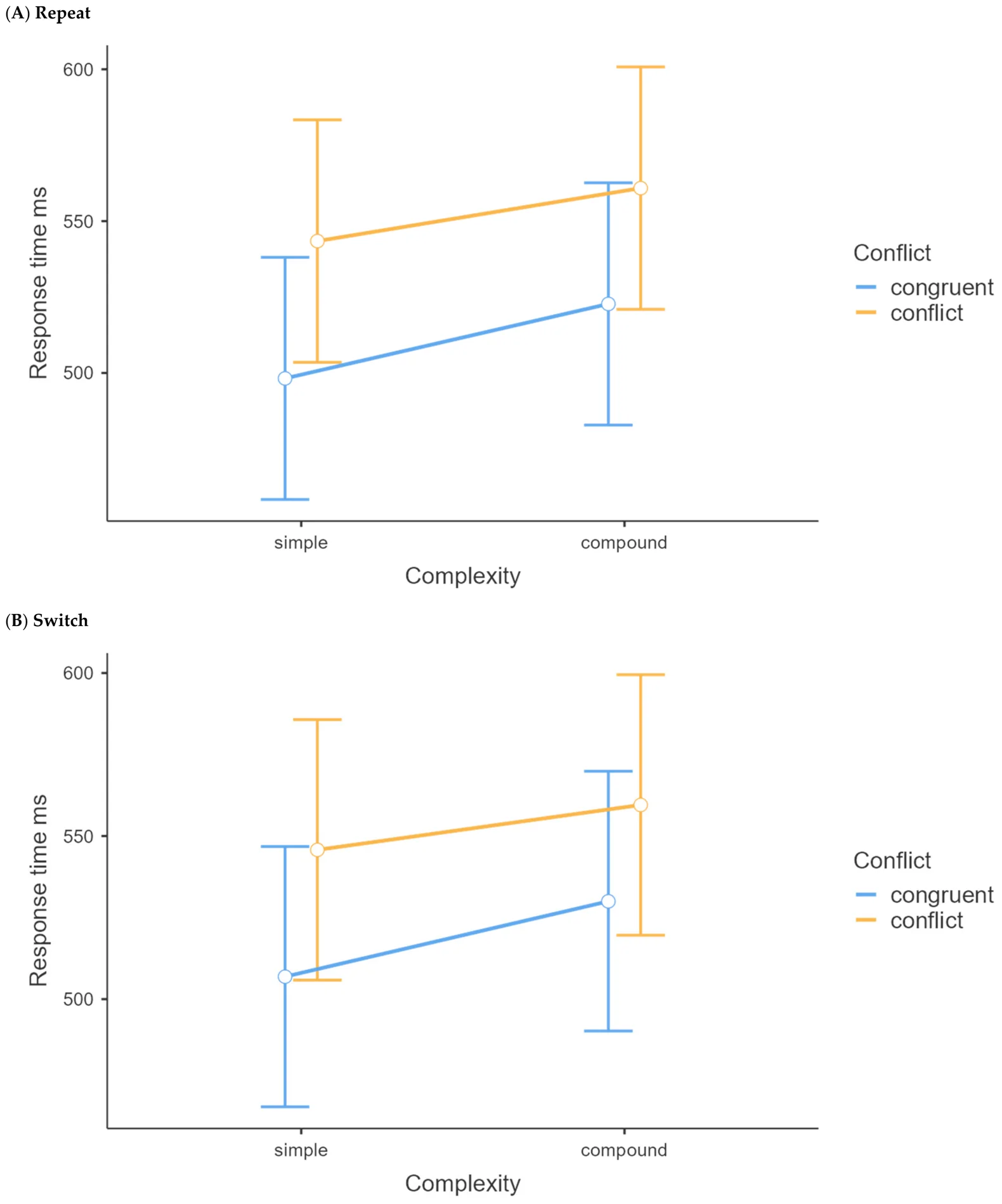
Model-estimated response times across conflict, complexity, and sequence conditions in Experiment 2. Error bars indicate 95% confidence intervals.

Taken together, the response-time results replicated the main costs of conflict and stimulus-response complexity from Experiment 1. Response times increased when relevant and irrelevant stimulus dimensions were incongruent and when the task involved compound rather than simple stimulus-response processing. In contrast, sequence-related slowing was weak, and the interaction tests did not reach significance. The model-estimated response-time pattern across conditions is shown in Table 7 and Figure 9.

#### 3.2.4. The Effect of Conflict, Complexity, and Sequence on Premotor Time and Motor Time

To determine whether the observed response-time effects emerged before or after the onset of measurable muscle activation, we decomposed response time into premotor and motor components. Premotor time was defined as the interval between stimulus onset and the onset of correct-hand EMG activation, whereas motor time was defined as the interval between correct-hand EMG onset and the overt button press. Both analyses were restricted to fully correct pure-correct trials with valid EMG onset and response markers. Two trials identified as outliers in the premotor-time box plot were excluded from the premotor-time analysis.

Premotor time was analyzed using a linear mixed-effects model with conflict, complexity, sequence, and their interactions as fixed effects and a random intercept for participant. The model was estimated on 13,788 trials from 47 participants. The fixed-effects structure was significant, χ^2^(7) = 282.64, *p* < 0.001. The model yielded a marginal R^2^ of 0.01 and a conditional R^2^ of 0.46.

Premotor times were longer in conflict than congruent trials, F(1, 13,736.85) = 199.60, *p* < 0.001 (estimated effect = 32.52 ms, 95% CI [28.00, 37.03]), and in compound than in simple trials, F(1, 13,734.16) = 73.32, *p* < 0.001 (estimated effect = 19.54 ms, 95% CI [15.07, 24.02]). The sequence effect and all interactions did not reach significance (all *p* values ≥ 0.141). Taken together, premotor times were longer in conflict and compound trials, whereas the analyses did not detect reliable sequence or interaction effects.

Motor times outside the range defined by the overall median ± 3 SD (median = 92.77 ms, SD = 34.12 ms) were excluded. Motor time was analyzed using the same fixed-effects structure and a random intercept for participant on 13,501 trials from 47 participants. The fixed-effects structure was significant, χ^2^(7) = 47.15, *p* < 0.001. Marginal R^2^ was 0.002, whereas conditional R^2^ was 0.56.

Motor time was longer in conflict than in congruent trials, F(1, 13,448.87) = 11.19, *p* < 0.001 (b = 1.17 ms, 95% CI [0.48, 1.85]), and in compound than in simple trials, F(1, 13,447.10) = 30.92, *p* < 0.001 (b = 1.93 ms, 95% CI [1.25, 2.60]). The sequence effect and all interactions did not reach significance (all *p* values ≥ 0.068). Taken together, motor-time differences associated with conflict and complexity were statistically significant but small in magnitude.

#### 3.2.5. Confidence in Partial-Error Occurrence as a Function of Motor Information and Task Conditions

The central analysis examined whether confidence in partial-error occurrence was related to EMG-derived characteristics of the incorrect motor activation and whether these relationships varied across task conditions. To address this question, we fitted a cumulative link mixed model (CLMM) with confidence ratings as the ordinal dependent variable. The model included incorrect-burst EMG surface, correction time, conflict, complexity, and sequence as fixed effects, together with all two-way interactions among task factors, interactions of incorrect-burst surface and correction time with each task factor, and the Conflict × Complexity × Sequence interaction. A random intercept was included for participants. Incorrect-burst surface and correction time were log10-transformed to reduce positive skew and subsequently standardized separately within each participant. The model was estimated on 1689 partial-error trials from 44 participants. The fixed-effects structure was significant, χ^2^(15) = 187.39, *p* < 0.001. Marginal and conditional R^2^ were 0.08 and 0.41, respectively. Incorrect EMG surface was a strong positive predictor of confidence. Larger incorrect-burst surface was associated with higher confidence that a partial error had occurred, b = 0.50, OR = 1.64, 95% CI [1.50, 1.80], *p* < 0.001. Correction time also positively predicted confidence, b = 0.30, OR = 1.35, 95% CI [1.23, 1.48], *p* < 0.001. Thus, confidence was related both to the magnitude of the incorrect EMG activation and to the time separating the initial incorrect activation from the subsequent correct activation. Partial-error confidence was lower in simple than in compound trials (estimated confidence = 2.66 vs. 2.94). This difference was statistically significant, b = −0.32, OR = 0.73, 95% CI [0.61, 0.87], *p* < 0.001. The main effects of conflict and sequence did not reach significance. The association between incorrect-burst surface and confidence was stronger in simple than in compound trials, b = 0.24, OR = 1.27, 95% CI [1.06, 1.52], *p* = 0.011. Confidence increased with incorrect-burst surface under both levels of complexity. The association between correction time and confidence was stronger in simple than in compound trials, b = 0.27, OR = 1.31, 95% CI [1.08, 1.59], *p* = 0.005. Correction time was positively associated with confidence in both compound trials (OR = 1.18, *p* = 0.011) and simple trials (OR = 1.54, *p* < 0.001). The remaining interactions did not reach significance (all *p* values ≥ 0.624).

We next fitted an additional model including RT as a covariate to test whether the associations between EMG-derived response-process characteristics and confidence could be accounted for by global response slowing. Before its inclusion, RT was log10-transformed and standardized separately within each participant in the same manner as the other continuous predictors. The RT-adjusted fixed-effects structure was significant, χ^2^(16) = 238.72, *p* < 0.001. Marginal and conditional R^2^ were 0.09 and 0.43, respectively. RT was a significant positive predictor of confidence, b = 0.37, OR = 1.45, 95% CI [1.32, 1.61], *p* < 0.001. Crucially, incorrect EMG surface remained a robust predictor after controlling for RT, b = 0.45, OR = 1.56, 95% CI [1.43, 1.72], *p* < 0.001. Correction time was attenuated but remained significant, b = 0.15, OR = 1.16, 95% CI [1.04, 1.28], *p* = 0.005. Partial-error confidence remained lower in simple than in compound trials after adjustment for RT. The complexity effect remained significant, b = −0.29, OR = 0.75, 95% CI [0.63, 0.90], *p* = 0.002. The association between incorrect-burst surface and confidence remained stronger in simple than in compound trials after adjustment for RT, b = 0.24, OR = 1.27, 95% CI [1.06, 1.53], *p* = 0.010. Confidence increased with incorrect-burst surface in both complexity conditions. The association between correction time and confidence remained stronger in simple than in compound trials, b = 0.24, OR = 1.27, 95% CI [1.05, 1.54], *p* = 0.012. After adjustment for RT, correction time predicted confidence in simple trials (OR = 1.31, *p* < 0.001), whereas the corresponding estimate in compound trials did not reach significance (OR = 1.03, *p* = 0.704).

Taken together, partial-error confidence was positively associated with incorrect-burst surface and correction time. The association with incorrect-burst surface remained robust after RT adjustment, whereas the correction-time association was attenuated and remained reliable in simple trials; the corresponding estimate in compound trials did not reach significance. Stimulus complexity moderated both relationships, with stronger associations in simple than in compound trials. Thus, the relationship between measurable characteristics of the partial-error event and confidence varied with task context.

#### 3.2.6. Metacognitive Sensitivity and Efficiency Analyses

Finally, we examined initial-motor-response sensitivity, metacognitive sensitivity, and metacognitive efficiency of partial-error judgments. Initial-motor-response sensitivity was indexed by d′, metacognitive sensitivity by meta-d′, and metacognitive efficiency by M-ratio. Estimates were obtained separately for each Conflict × Complexity condition.

In the binary SDT representation, the stimulus class was the response hand required by the colour stimulus and the response class was the hand of the first detected EMG burst. Consequently, pure-correct and partial-error trials differed in the hand of the initial motor response. In pure-correct trials, the initial response occurred in the stimulus-required hand, whereas in partial-error trials, it occurred in the opposite hand and was subsequently corrected before the overt response.

The analysis included 42 participants. Initial-motor-response sensitivity was higher in congruent than in conflict trials, F(1, 41) = 13.88, *p* < 0.001, ηp^2^ = 0.25, and higher in compound than in simple trials, F(1, 41) = 11.47, *p* = 0.002, ηp^2^ = 0.22. The Conflict × Complexity interaction did not reach significance, F(1, 41) = 0.41, *p* = 0.523.

**Figure 10:**
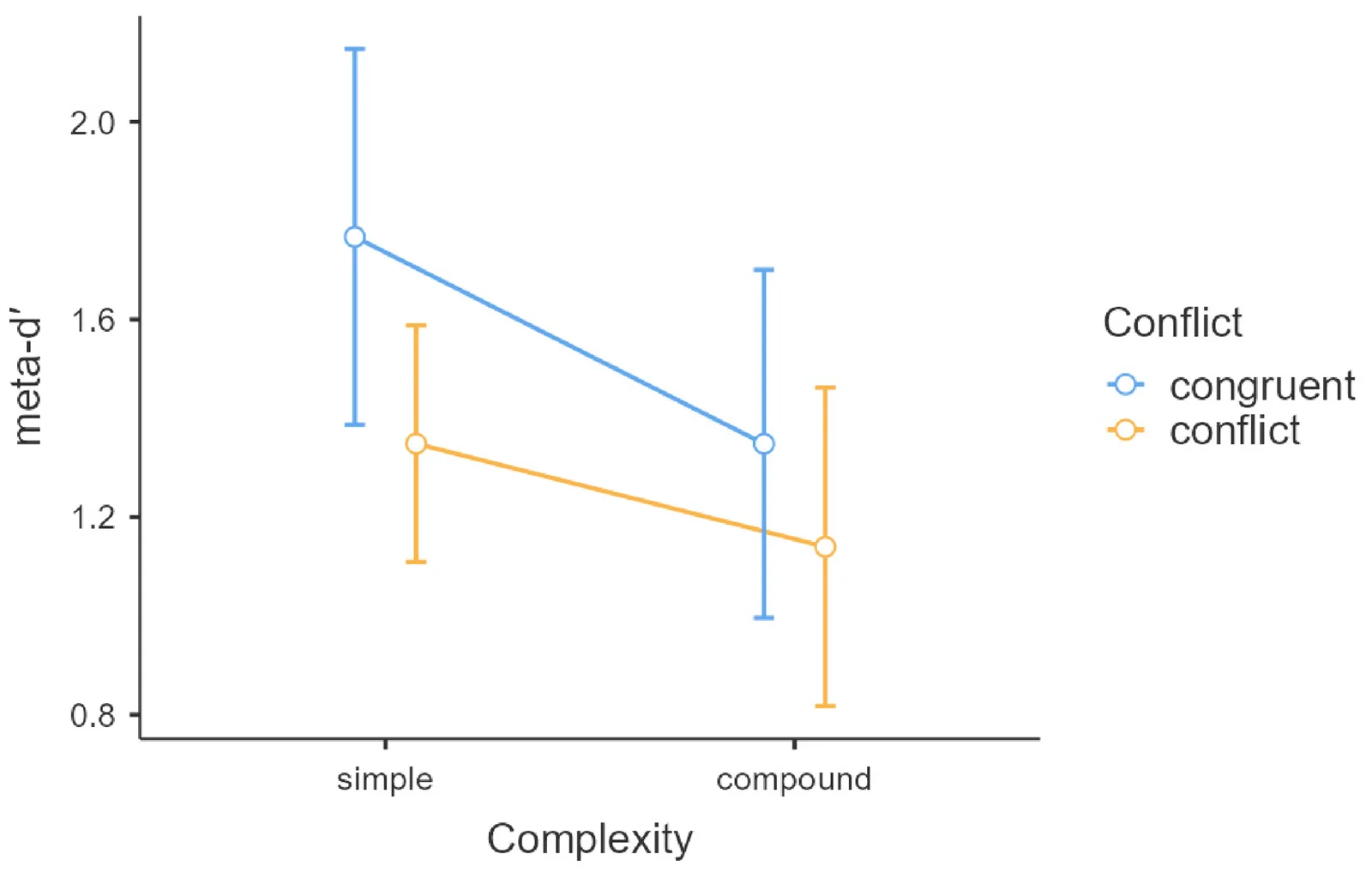
Marginal means of metacognitive sensitivity (meta-d′) as a function of conflict and complexity in Experiment 2.

We next examined metacognitive sensitivity, defined here as the extent to which the six graded confidence levels discriminated between pure-correct and partial-error trials. Meta-d′ was higher in congruent than in conflict trials, F(1, 41) = 10.70, *p* = 0.002, ηp^2^ = 0.21, and higher in simple than in compound trials, F(1, 41) = 8.51, *p* = 0.006, ηp^2^ = 0.17. The Conflict × Complexity interaction did not reach significance, F(1, 41) = 1.34, *p* = 0.254. Thus, both conflict and stimulus complexity reduced metacognitive sensitivity to partial errors, while the interaction term did not reach significance. The pattern of meta-d′ across conflict and complexity conditions is shown in Table 8 and Figure 10.

We next examined metacognitive efficiency using M-ratio. M-ratio was higher in simple than in compound trials, F(1, 41) = 13.45, *p* < 0.001, ηp^2^ = 0.25. The conflict effect, F(1, 41) = 1.28, *p* = 0.264, and the Conflict × Complexity interaction, F(1, 41) = 0.03, *p* = 0.872, did not reach significance.

**Figure 11:**
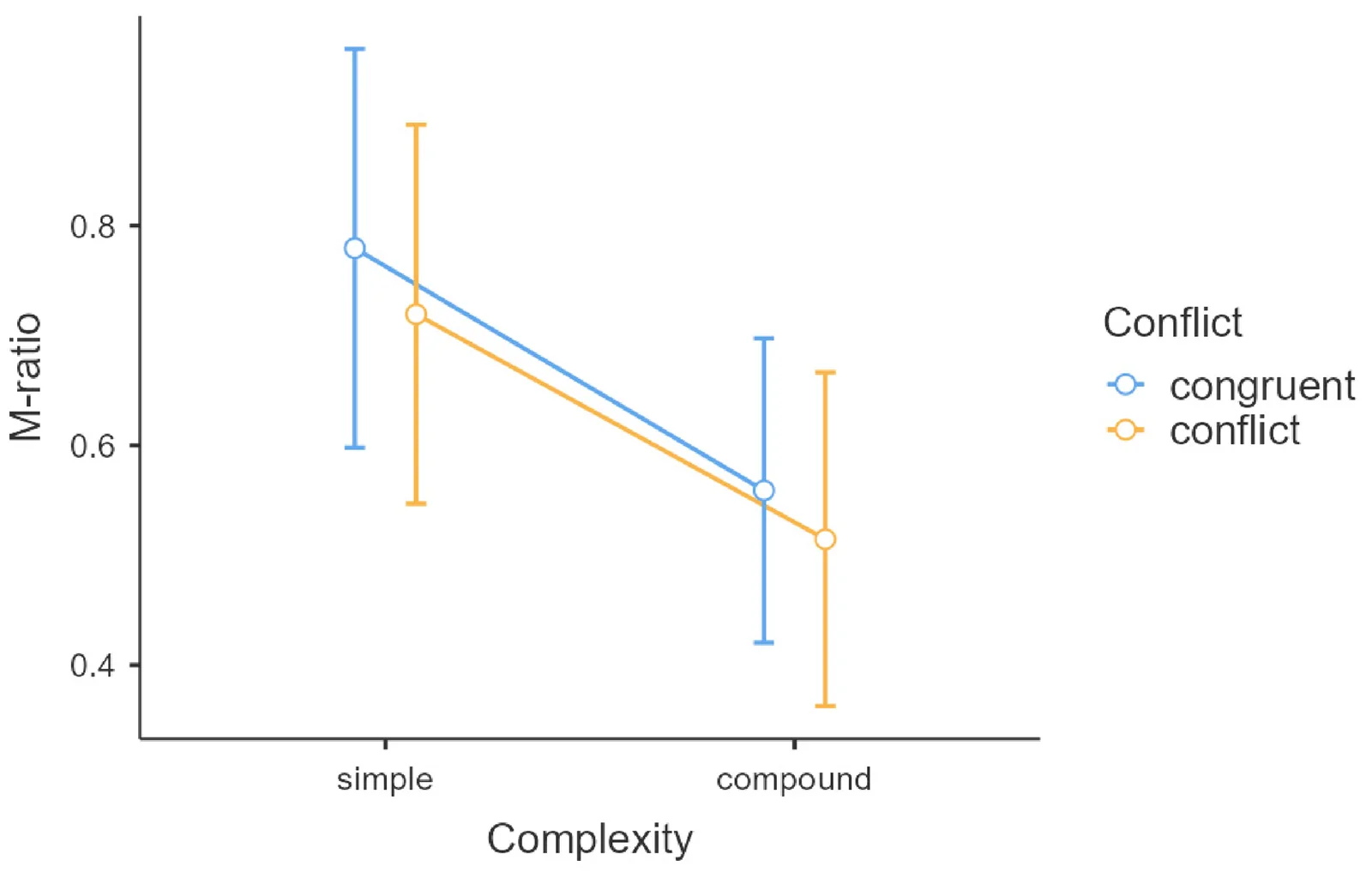
Estimated marginal means of metacognitive efficiency (M-ratio) as a function of conflict and complexity in Experiment 2.

Taken together, conflict and complexity had different effects on first-order motor and metacognitive measures. Conflict was associated with a higher incidence of partial errors, lower d′, and lower meta-d′, whereas the conflict effect on M-ratio did not reach significance. Complexity showed a different pattern: partial errors were less frequent and d′ was higher in compound than in simple trials, whereas both meta-d′ and M-ratio were lower in compound trials. The reduction in M-ratio for compound relative to simple trials is shown in Table 9 and Figure 11.

### 3.3. Interim Discussion of Experiment 2

In Experiment 2, we investigated whether the cognitive-control demands imposed by conflict, stimulus complexity, and trial sequence affect task performance, reported confidence in partial errors, the relationship between confidence and motor activations, and metacognitive monitoring of partial errors.

Similar to Experiment 1, we observed behavioural costs of conflict: response accuracy was lower when task-relevant and task-irrelevant stimulus dimensions were incongruent. However, the strength of the influence of conflict on task accuracy differed between complexity and sequence conditions, and it was largest when a compound stimulus followed a simple stimulus, compared to when a simple stimulus followed a compound stimulus.

Although complexity and sequence did not produce main effects on response accuracy, response times were clearly sensitive to conflict and complexity. These effects are consistent with increased interference and stimulus-processing demands, but we found no reliable evidence that complexity modulated the conflict effect on response time.

The results showed that increased control demands were expressed primarily in the premotor segment of responding. Premotor time was prolonged by both conflict and complexity, indicating that incongruent and compound trials delayed the initiation of response-related muscle activity. The conflict effect is consistent with conflict-monitoring and activation–suppression accounts, according to which incongruent stimuli activate competing response tendencies that require additional control before the selected action can be released into the motor system [56,57,58]. The complexity effect, in turn, is consistent with response-programming accounts suggesting that more complex response requirements increase the time needed to prepare the motor command before execution [59,60].

Motor time showed the same general direction, with longer post-EMG intervals under conflict and for compound stimuli, but these effects were very small in absolute magnitude. Thus, most of the temporal cost associated with conflict and complexity arose before EMG-defined response initiation. This pattern suggests that control demands primarily affected stimulus evaluation, response selection, and motor-command preparation rather than the peripheral implementation of an already initiated response. Nevertheless, motor time should not be treated as a purely peripheral measure, because post-EMG execution intervals may also be influenced by response force, movement organization, and late decisional constraints [61,62].

The analyses of confidence in partial-error occurrence showed that the level of reported confidence was related to motor-level information. As expected, confidence that a partial error had occurred increased when the incorrect EMG activation was stronger and when correction time was longer. This pattern is consistent with previous work showing that partial-error detection and confidence are associated with properties of incorrect motor activation, especially its amplitude or surface and the time required for correction [12,13,25].

These results extend previous evidence linking partial-error characteristics to subjective detection and confidence by showing that the associations between partial-error characteristics and confidence varied across task conditions. Specifically, both incorrect-burst surface and correction time showed weaker associations with confidence during compound processing. The association involving incorrect-burst surface remained robust after adjustment for overall response time, whereas the association involving correction time was attenuated and remained reliable only in simple trials. The additional processing required in compound trials may therefore have altered how information about the preceding response event contributed to retrospective confidence judgments. At the same time, partial-error confidence was lower in simple than in compound trials, suggesting that task complexity affected both the overall level of confidence and its relationship with response-process characteristics. However, these correlational associations may also reflect shared influences on partial-error characteristics and confidence judgments, such as response competition, experienced task difficulty, or other underlying processes.

The exploratory SDT analyses provided a complementary characterization of this pattern. Conflict was associated with lower initial-motor-response sensitivity and lower meta-d′, whereas complexity was associated with higher initial-motor-response sensitivity but lower meta-d′ and M-ratio. Together with the trial-level analyses, these findings indicate that cognitive-control conditions were related both to the occurrence of partial errors and to the relationship between measurable characteristics of those events and their subsequent metacognitive evaluation.

## 4. General Discussion

Across two experiments, we examined how cognitive-control demands influenced retrospective metacognitive judgments directed at different aspects of performance. In Experiment 1, participants evaluated the correctness of the completed colour-plus-line response sequence. In Experiment 2, they evaluated whether a partial error had occurred during preparation of the colour response. The two experiments therefore addressed metacognitive evaluation at different levels of the response episode: the completed behavioural outcome and an objectively identifiable event within the unfolding response trajectory.

Conflict consistently reduced confidence in response correctness. Complexity and sequence also influenced confidence through their interaction. These effects can be considered in relation to the specific types of task demands involved. Firstly, the presence of conflicting stimuli reduced confidence even though only correct responses were compared. This suggests that conflict does not merely impair objective performance; it also changes how participants evaluate their responses. This result is coherent with some views on confidence assessments as based on multiple sources of information available during and after the decision process, including decision and motor evidence, response fluency, and cognitive conflict (e.g., [2,27,41,63,64,65,66]). In this framework, detection of response conflict may increase uncertainty: when competing response tendencies are activated, the selected response may feel less accurate even if it is ultimately correct. Secondly, sequence and complexity interacted in their effects on confidence. Confidence was higher in simple-repeated than in simple-switched trials, whereas it was higher in compound-switched than in compound-repeated trials. We assumed that responding to compound stimuli requires updating of working memory and this additional strain on cognitive resources could reduce response confidence. One potentially informative feature of this interaction was the relatively low confidence observed in compound-repeated trials compared with simple-repeated trials. This means that confidence was lower when a compound trial was preceded by another compound trial, compared to when a simple trial was preceded by another simple trial. In our interpretation, this may reflect the continued requirement to update working memory across consecutive compound trials: when participants have to repeatedly process complex information, they may become less certain about the correctness of their responses.

In Experiment 2, partial-error confidence was lower in simple than in compound trials, while the relationships between confidence and both incorrect-burst surface and correction time were weaker in compound trials. Previous EMG studies established that characteristics of incorrect motor activation and correction timing are associated with partial-error detection and confidence. The present study extends this work by showing that these relationships vary with cognitive-control context. Thus, additional processing demands were associated not only with the level of reported confidence but also with the relationship between measurable characteristics of the partial-error event and its subsequent evaluation.

Interestingly, we did not detect a significant effect of conflict on partial-error confidence, despite conflict being a plausible candidate for influencing confidence in partial-error events. We do not interpret this absence of evidence as evidence of absence. It is nevertheless noteworthy that fewer effects of task demands on performance measures were observed when confidence in partial errors was assessed. This pattern may reflect differences between Experiment 1, in which participants reported confidence in response correctness immediately after responding, and Experiment 2, in which participants monitored their response process for partial errors and reported confidence retrospectively at the end of the trial. Because these procedures differed in both judgement target and task requirements, caution is warranted when comparing the patterns of effects across experiments.

The results of both experiments suggest that metacognitive sensitivity is also affected by task demands. When confidence in response accuracy was measured, metacognitive sensitivity (meta-*d′*) was lower in compound than in simple trials, and this reduction was strongest when compound stimuli were processed under conflict. Importantly, the same complexity effect and its interaction with conflict were observed when trials with an incorrect line-position response were excluded, strengthening this result across alternative first-order specifications. When confidence in partial-error occurrence was measured, meta-*d′* was lower under both conflict and complexity. Thus, task demands affected the correspondence between objective response-related information and metacognitive judgments in both experiments, although the precise pattern depended on the monitored outcome.

When confidence in partial-error occurrence was measured, complexity also affected metacognitive efficiency: M-ratio was lower in compound than in simple trials. Importantly, partial errors themselves were less frequent in compound trials, resulting in higher first-order *d′*. Because partial-error trials corresponded to initial-motor-response mismatches in this adapted representation, differences in M-ratio should be interpreted relative to the corresponding differences in initial-motor-response sensitivity. Nevertheless, the lower meta-*d′* and M-ratio converged with the trial-level finding that the relation between motor signals and confidence was weaker in compound trials. This pattern is consistent with Maniscalco and Lau’s [35] finding that active manipulation of working-memory contents can impair metacognitive monitoring.

Taken together, the trial-level and SDT findings are consistent with the proposal that working memory includes a motor component supporting the short-term maintenance and use of action-related information [67].

A different pattern emerged for confidence in response correctness. In Experiment 1, conflict substantially reduced first-order *d′* without producing an overall reduction in meta-*d′*, and M-ratio was consequently higher under conflict than under congruent conditions. This overall conflict-related increase in M-ratio was retained when trials with an incorrect line-position response were excluded, although its magnitude then depended on complexity. Because M-ratio expresses meta-*d′* relative to first-order sensitivity, these results indicate that first-order sensitivity was more strongly affected by conflict than metacognitive sensitivity; they should not be interpreted as an absolute enhancement of metacognitive monitoring [41,48,68].

Taken together, the present findings suggest that metacognition may be linked to different, partly separable functions of cognitive control rather than to a single global difficulty signal. Interference provided a reliable cue that the response was less certain: it reduced performance and confidence in response correctness, while its effects on metacognitive sensitivity depended on the monitored outcome. Complexity, in turn, affected both metacognitive sensitivity and the relationship between motor evidence and confidence. These effects are consistent with the possibility that metacognitive judgments require additional working-memory resources when they depend on response-related information. In line with this reasoning, confidence judgments may rely on multiple diagnostic cues associated with different aspects of cognitive-control engagement.

The distinction between final behavioural outcome and response trajectory also suggests a potential practical direction. In the present study, partial errors differentiated behaviourally correct responses according to whether competing activation reached the motor level before correction. Because metacognitive monitoring is thought to support the regulation and adjustment of ongoing behaviour, information about such events may be informative before overt performance deteriorates. Repeated increases in partial-error occurrence, changes in correction dynamics, or increased confidence that such events have occurred could potentially signal emerging difficulties in response control while successful performance is still maintained. These possibilities may be relevant to contexts in which behavioural accuracy alone provides limited information about emerging performance difficulties, including human–machine interaction, operator monitoring, and other monitoring-intensive environments. However, the present study did not evaluate real-time prediction, prospective monitoring of performance, or adaptive intervention. Establishing practical utility will therefore require prospective studies and validation in more naturalistic settings.

Several limitations constrain the interpretation of these findings. First, conflict, complexity, and sequence were designed to vary demands associated with interference control, working-memory updating, and shifting, but they were not process-pure measures of those executive functions. The results should therefore be interpreted primarily in terms of the experimental factors. Second, both samples consisted predominantly of young adults performing discrete manual responses in a controlled modified Stroop task. The extent to which the present findings generalize to other populations, response modalities, and more naturalistic settings therefore remains to be established. Third, the signal-detection measures were adapted to tasks more complex than the simple detection and discrimination paradigms for which meta-*d′* is most commonly used. In Experiment 1, first-order correctness was defined for the complete colour-plus-line response sequence, corresponding to the outcome evaluated by confidence. Because this representation treated isolated line-position errors as first-order errors, we repeated the analyses after excluding these trials; the principal meta-*d′* findings and the overall conflict effect on M-ratio were retained. In Experiment 2, first-order performance was defined by whether initial motor activation occurred in the task-required or incorrect response hand. Partial errors were less frequent in compound trials and therefore produced higher *d′*, which should be considered when interpreting the corresponding meta-*d′* and M-ratio effects. Estimates from the two experiments consequently refer to different first-order representations and should not be compared quantitatively [41,68]. A further limitation of the present study concerns ceiling effects and the restricted range of performance and confidence measures, particularly in Experiment 1. Model-estimated accuracy ranged from 0.92 to 0.99 across conditions, and confidence ratings were concentrated near the upper end of the 13-point scale (12.43–12.65). The conflict-related difference in confidence was 0.06 points on the 13-point scale. Although this difference was small in absolute terms, confidence was nevertheless reliably lower in conflict than in congruent trials. Finally, handedness was not formally assessed. However, left- and right-hand responses occurred with equal frequency across conditions.

## 5. Conclusions

Cognitive-control demands were associated with both confidence and response dynamics. Conflict was associated with impaired overt performance and a small reduction in confidence in the correctness of completed responses. By contrast, confidence that a partial error had occurred was associated with the magnitude and timing of the partial-error event, and these associations were weaker during compound processing. Together, these findings show that the relationship between measurable response-process characteristics and confidence is shaped by the cognitive-control demands under which decisions are made.

## Figures and Tables

**Table 1:** Model-estimated response accuracy across conflict, complexity, and sequence conditions in Experiment 1.

					95% Confidence Intervals	
Conflict	Complexity	Sequence	Mean	SE	Lower	Upper
congruent	simple	repeat	0.99	0.00	0.98	0.99
congruent	simple	switch	0.97	0.00	0.96	0.98
congruent	compound	repeat	0.95	0.01	0.94	0.96
congruent	compound	switch	0.95	0.01	0.94	0.96
conflict	simple	repeat	0.97	0.00	0.96	0.98
conflict	simple	switch	0.94	0.01	0.92	0.95
conflict	compound	repeat	0.92	0.01	0.90	0.94
conflict	compound	switch	0.92	0.01	0.90	0.93

**Table 2:** Model-estimated response times as a function of conflict, complexity, and sequence in Experiment 1.

					95% Confidence Intervals	
Conflict	Complexity	Sequence	Mean	SE	Lower	Upper
congruent	simple	repeat	568.29	9.51	549.64	586.94
congruent	simple	switch	574.75	9.09	556.94	592.57
congruent	compound	repeat	680.51	9.12	662.63	698.39
congruent	compound	switch	639.19	8.89	621.77	656.61
conflict	simple	repeat	590.14	10.02	570.50	609.77
conflict	simple	switch	614.00	9.66	595.07	632.92
conflict	compound	repeat	657.29	10.12	637.46	677.12
conflict	compound	switch	672.24	9.95	652.73	691.76

**Table 3:** Model-estimated confidence in response correctness as a function of conflict, complexity, and sequence in Experiment 1.

					95% Confidence Intervals	
Conflict	Complexity	Sequence	Mean	SE	Lower	Upper
congruent	simple	repeat	12.65	0.08	12.50	12.80
congruent	simple	switch	12.51	0.08	12.36	12.66
congruent	compound	repeat	12.49	0.08	12.34	12.64
congruent	compound	switch	12.57	0.08	12.42	12.72
conflict	simple	repeat	12.59	0.08	12.44	12.74
conflict	simple	switch	12.45	0.08	12.30	12.60
conflict	compound	repeat	12.43	0.08	12.28	12.58
conflict	compound	switch	12.52	0.08	12.37	12.67

**Table 4:** Estimated marginal means of metacognitive sensitivity as a function of conflict and complexity in Experiment 1.

				95% Confidence Interval	
Conflict	Complexity	Mean	SE	Lower	Upper
congruent	simple	4.31	0.10	4.10	4.51
	compound	3.97	0.16	3.66	4.29
conflict	simple	4.37	0.11	4.14	4.59
	compound	3.67	0.15	3.37	3.97

**Table 5:** Estimated marginal means of metacognitive efficiency as a function of conflict and complexity in Experiment 1.

				95% Confidence Interval	
Conflict	Complexity	Mean	SE	Lower	Upper
congruent	simple	1.19	0.03	1.12	1.25
	compound	1.21	0.07	1.07	1.36
conflict	simple	1.37	0.04	1.29	1.45
	compound	1.39	0.06	1.28	1.51

**Table 6:** Model-estimated response accuracy across conflict, complexity, and sequence conditions in Experiment 2.

					95% Confidence Intervals	
Conflict	Complexity	Sequence	Mean	SE	Lower	Upper
congruent	simple	repeat	0.90	0.01	0.87	0.92
congruent	simple	switch	0.88	0.02	0.84	0.91
congruent	compound	repeat	0.89	0.02	0.85	0.91
congruent	compound	switch	0.90	0.01	0.87	0.92
conflict	simple	repeat	0.84	0.02	0.79	0.87
conflict	simple	switch	0.83	0.02	0.79	0.87
conflict	compound	repeat	0.83	0.02	0.79	0.87
conflict	compound	switch	0.83	0.02	0.78	0.87

**Table 7:** Model-estimated response times across task conditions in Experiment 2. Estimated marginal means are expressed in milliseconds.

						95% Confidence Intervals	
Conflict	Complexity	Sequence	Mean	SE	df	Lower	Upper
congruent	simple	repeat	498.2	19.82	47.84	458.3	538.1
congruent	simple	switch	506.9	19.85	48.07	467.0	546.8
congruent	compound	repeat	522.7	19.83	47.93	482.9	562.6
congruent	compound	switch	530.0	19.84	48.01	490.1	569.9
conflict	simple	repeat	543.4	19.85	48.12	503.5	583.4
conflict	simple	switch	545.8	19.87	48.24	505.8	585.7
conflict	compound	repeat	560.9	19.86	48.17	520.9	600.8
conflict	compound	switch	559.5	19.87	48.28	519.6	599.5

**Table 8:** Estimated marginal means of metacognitive sensitivity (meta-d′) as a function of conflict and complexity in Experiment 2.

				95% Confidence Interval	
Complexity	Conflict	Mean	SE	Lower	Upper
simple	congruent	1.77	0.19	1.39	2.15
	conflict	1.35	0.12	1.11	1.59
compound	congruent	1.35	0.17	1.00	1.70
	conflict	1.14	0.16	0.82	1.46

**Table 9:** Estimated marginal means of metacognitive efficiency (M-ratio) as a function of conflict and complexity in Experiment 2.

				95% Confidence Interval	
Complexity	Conflict	Mean	SE	Lower	Upper
simple	congruent	0.78	0.09	0.60	0.96
	conflict	0.72	0.09	0.55	0.89
compound	congruent	0.56	0.07	0.42	0.70
	conflict	0.51	0.08	0.36	0.67

## Data Availability

The original data supporting the findings of this study are available in the Open Science Framework (OSF) via the following anonymized view-only link: https://osf.io/mq5b8/overview?view_only=c1a190ebc24a45d5b6a0da86c1669c48 (accessed on 6 September 2026).

## Supplementary Materials

The following supporting information can be downloaded at: https://www.mdpi.com/article/10.3390/brainsci16090975/s1, Table S1: Model-estimated confidence in response correctness as a function of Conflict, Complexity, and Sequence in Experiment 1; Table S2: Estimated marginal means of metacognitive sensitivity as a function of Conflict and Complexity in Experiment 1; Table S3: Estimated marginal means of metacognitive efficiency as a function of Conflict and Complexity in Experiment 1; Table S4: Model-estimated response accuracy across Conflict, Complexity, and Sequence conditions in Experiment 2; Table S5: Model-estimated response times across task conditions in Experiment 2. Estimated marginal means are expressed in milliseconds. Confidence intervals represent 95% confidence intervals; Table S6: Estimated marginal means of metacognitive sensitivity (meta-d′) as a function of Conflict and Complexity in Experiment 2; Table S7: Estimated marginal means of metacognitive efficiency (M-ratio) as a function of Conflict and Complexity in Experiment 2; Figure S1: Marginal means of metacognitive sensitivity (meta-d′) as a function of Conflict and Complexity in Experiment 2; Figure S2: Estimated marginal means of metacognitive efficiency (M-ratio) as a function of Conflict and Complexity in Experiment 2.
