# Supplementary material for: Cognitive Control and Metacognition: The Effect of Interference, Working-Memory Load, and Shifting on Confidence in Response Correctness and Partial Error
Source: Brain Sci. 2026 Sep 15;16(9):975. doi: 10.3390/brainsci16090975 (PMC13604749; doi:10.3390/brainsci16090975)
Supplement: Supplementary file 1 [file brainsci-16-00975-s001.zip › brainsci-4497070-supplementary.pdf]

Supplementary Materials

S1. Supplementary Results: Experiment 1
S1.1. Accuracy
S1.2. Response Times
S1.3. Confidence Judgments
S1.4. Exploratory Signal Detection Analyses

S2. Supplementary Results: Experiment 2
S2.1. Accuracy
S2.2. Partial-Error Occurrence
S2.3. Response Times
S2.4. Premotor Time
S2.5. Motor Time
S2.6. Partial-Error Confidence
S2.7. Exploratory Signal Detection Analyses

S1.1. Accuracy

Firstly, we examined whether accuracy in the modified Stroop task was decreased by conflict, stimulus complexity, and sequence. Accuracy was analyzed using a generalized linear mixed-effects model with a binomial distribution and a logit link function. The model included Conflict, Complexity, Sequence, and their interactions as fixed effects, with a random intercept for participant. The model was fitted to 29,502 trials from 66 participants.

The fixed-effects structure was significant, marginal $R^2 = .08$, $\chi^2(7) = 426.56$, $p < .001$. Conditional R^2 was .23 after including participant-level variability. The random intercept variance was 0.67, SD = 0.82, ICC = .17.

The model revealed significant main effects of Conflict, $\chi^2(1) = 142.86$, $p < .001$, Complexity, $\chi^2(1) = 212.45$, $p < .001$, and Sequence, $\chi^2(1) = 48.73$, $p < .001$. Overall, participants were more accurate in congruent trials, M = .97, SE = .00, 95% CI [.96, .97], than in conflict trials, M = .94, SE = .01, 95% CI [.93, .95], OR = 1.93, SE = 0.11, 95% CI [1.74, 2.15], z = 11.95, $p < .001$. Accuracy was also higher in simple trials, M = .97, SE = .00, 95% CI [.96,

.98], than in compound trials, M = .94, SE = .01, 95% CI [.92, .95], OR = 2.23, SE = 0.12, 95% CI [2.01, 2.49], z = 14.58, $p < .001$. Finally, participants were more accurate in repeated trials, M = .96, SE = .00, 95% CI [.96, .97], than in switched trials, M = .95, SE = .01, 95% CI [.94, .96], OR = 1.47, SE = 0.08, 95% CI [1.32, 1.64], z = 6.98, $p < .001$. These effects confirm that accuracy was affected by all three experimental manipulations of the targeted cognitive-control components.

The model revealed a significant Conflict × Complexity interaction, $\chi^2(1) = 8.48$, $p = .004$. The conflict effect was significant for both simple and compound trials, but it was larger for simple trials. Participants were more accurate in congruent-simple trials, M = .98, SE = .00, 95% CI [.97, .98], than in conflict-simple trials, M = .96, SE = .00, 95% CI [.95, .96], OR = 2.27, SE = 0.21, 95% CI [1.80, 2.87], z = 9.01, $p < .001$. The conflict effect was also significant for compound trials, but it was smaller: participants were more accurate in congruent-compound trials, M = .95, SE = .01, 95% CI [.94, .96], than in conflict-compound trials, M = .92, SE = .01, 95% CI [.90, .93], OR = 1.65, SE = 0.10, 95% CI [1.40, 1.93], z = 8.02, $p < .001$.

The model also revealed a significant Complexity × Sequence interaction, $\chi^2(1) = 39.83$, $p < .001$. The sequence effect was significant for simple trials, but not for compound trials. Participants were less accurate in simple-switched trials, that is, simple trials following compound trials, M = .96, SE = .00, 95% CI [.95, .97], than in simple-repeated trials, that is, simple trials following simple trials, M = .98, SE = .00, 95% CI [.97, .98], OR = 2.08, SE = 0.19, 95% CI [1.65, 2.63], z = 8.05, $p < .001$. In contrast, accuracy did not differ between compound-switched trials, that is, compound trials following simple trials, M = .94, SE = .01, 95% CI [.92, .95], and compound-repeated trials, that is, compound trials following compound trials, M = .94, SE = .01, 95% CI [.92, .95], OR = 1.04, SE = 0.06, 95% CI [0.88, 1.22], z = 0.60, $p = .550$, Tukey-adjusted $p = .933$. Thus, the sequence effect was larger for simple than compound trials. The Conflict × Sequence interaction was not significant, $\chi^2(1) = 0.10$, $p = .753$. The three-way Conflict × Complexity × Sequence interaction was also not significant, $\chi^2(1) = 0.77$, $p = .381$.

In summary, participants were less accurate in conflict than congruent trials, in compound than simple trials, and in

switched than repeated trials. However, these effects were qualified by two interactions. First, the conflict effect was larger for simple than compound trials. Second, the sequence effect was larger for simple than compound trials: accuracy was lower in simple-switched trials than in simple-repeated trials, whereas compound-switched and compound-repeated trials did not differ. The Conflict × Sequence interaction was not significant.

S1.2. Response Times

Next, we examined whether response times in the modified Stroop task were modulated by conflict, stimulus complexity, and sequence. Response times were analyzed using a linear mixed-effects model with Conflict, Complexity, Sequence, all two-way interactions, and the three-way interaction as fixed effects, with random intercepts for participants. The model converged successfully and was fitted to 26,787 observations from 66 participants. The model yielded a marginal R^2 of .05 and a conditional R^2 of .21. Variability in baseline response speed across participants was reflected in the random-intercept estimates, variance = 0.01, *SD* = 0.07, ICC = .16. The model was estimated using the bobyqa optimizer and Satterthwaite approximation for degrees of freedom.

The omnibus tests revealed a significant main effect of Conflict, $F(1, 26714.42) = 83.50$, $p < .001$. Participants responded more slowly in conflict trials, *M* = 630 ms, *SE* = 10, 95% CI [620, 650], than in congruent trials, *M* = 620 ms, *SE* = 10, 95% CI [600, 630], *b* = 20 ms, 95% CI [10, 20], $t(26714.42) = 9.14$, $p < .001$. Response times were also strongly modulated by stimulus complexity, $F(1, 26715.31) = 1507.79$, $p < .001$. Participants responded more slowly to compound trials, *M* = 660 ms, *SE* = 10, 95% CI [650, 680], than to simple trials, *M* = 590 ms, *SE* = 10, 95% CI [570, 600], *b* = 80 ms, 95% CI [70, 80], $t(26715.31) = 38.83$, $p < .001$. The main effect of Sequence was not significant, $F(1, 26715.22) = 0.29$, $p = .592$. Response times did not differ reliably between repeated trials, *M* = 620 ms, *SE* = 10, 95% CI [610, 640], and switched trials, *M* = 630 ms, *SE* = 10, 95% CI [610, 640], when averaged across Conflict and Complexity.

The model revealed significant Conflict × Sequence, $F(1, 26714.85) = 88.52$, $p < .001$, Conflict × Complexity, $F(1, 26714.24) = 42.10$, $p < .001$, and Sequence × Complexity interactions, $F(1, 26714.28) = 54.26$, $p < .001$. These two-way interactions were further qualified by a significant Conflict × Sequence × Complexity interaction, $F(1, 26715.07) = 24.05$, $p < .001$. We therefore decomposed the three-way interaction by examining conflict and complexity effects separately in repeated and switched trials.

In repeated trials, the conflict effect depended on stimulus complexity. For simple-repeated trials, that is, simple trials following simple trials, participants responded more slowly in conflict-simple-repeated trials, $M = 590$ ms, $SE = 10$, 95% CI [570, 610], than in congruent-simple-repeated trials, $M = 570$ ms, $SE = 10$, 95% CI [550, 590], difference = 20 ms, $z = 5.67$, $p < .001$. For compound-repeated trials, that is, compound trials following compound trials, the direction of the conflict effect was reversed: participants responded more slowly in congruent-compound-repeated trials, $M = 680$ ms, $SE = 10$, 95% CI [660, 700], than in conflict-compound-repeated trials, $M = 660$ ms, $SE = 10$, 95% CI [640, 680], difference = 20 ms, $z = 5.73$, $p < .001$.

In repeated trials, the complexity effect was significant at both levels of Conflict, but it was larger in congruent than conflict trials. In congruent-repeated trials, participants responded more slowly in compound-repeated trials, that is, compound trials following compound trials, than in simple-repeated trials, that is, simple trials following simple trials, difference = 110 ms, $z = 28.97$, $p < .001$. In conflict-repeated trials, participants also responded more slowly in compound-repeated than simple-repeated trials, but this effect was smaller, difference = 70 ms, $z = 17.31$, $p < .001$.

In switched trials, conflict increased response times for both simple and compound stimuli. For simple-switched trials, that is, simple trials following compound trials, participants responded more slowly in conflict-simple-switched trials, $M = 610$ ms, $SE = 10$, 95% CI [600, 630], than in congruent-simple-switched trials, $M = 570$ ms, $SE = 10$, 95% CI [560, 590], difference = 40 ms, $z = 10.16$, $p < .001$. For compound-switched trials, that is, compound trials following simple trials, participants also responded more slowly in conflict-compound-switched trials, M

= 670 ms, *SE* = 10, 95% CI [650, 690], than in congruent-compound-switched trials, *M* = 640 ms, *SE* = 10, 95% CI [620, 660], difference = 30 ms, $z = 8.40$, $p < .001$. Thus, in switched trials, the conflict effect was significant for both simple and compound stimuli and was slightly larger for simple than compound trials.

The complexity effect was also significant in switched trials. In congruent-switched trials, participants responded more slowly in compound-switched trials, that is, compound trials following simple trials, than in simple-switched trials, that is, simple trials following compound trials, difference = 60 ms, $z = 16.79$, $p < .001$. In conflict-switched trials, participants also responded more slowly in compound-switched than simple-switched trials, difference = 60 ms, $z = 14.72$, $p < .001$. Thus, in switched trials, the complexity effect was comparable across congruent and conflict trials.

Finally, we examined the sequence effect within each Conflict × Complexity combination. In congruent-simple trials, response times did not differ reliably between simple-switched trials, that is, simple trials following compound trials, and simple-repeated trials, that is, simple trials following simple trials, difference = 10 ms, $z = 1.74$, $p = .082$, Tukey-adjusted $p = .660$. In conflict-simple trials, participants responded more slowly in simple-switched than simple-repeated trials, difference = 20 ms, $z = 6.22$, $p < .001$. In congruent-compound trials, the sequence effect was reversed: participants responded faster in compound-switched trials, that is, compound trials following simple trials, than in compound-repeated trials, that is, compound trials following compound trials, difference = 40 ms, $z = 10.53$, $p < .001$. In conflict-compound trials, participants responded more slowly in compound-switched than compound-repeated trials, difference = 10 ms, $z = 3.65$, $p < .001$, Tukey-adjusted $p = .006$.

In summary, response times were longer in conflict than congruent trials and in compound than simple trials, whereas the main effect of Sequence was not significant. However, the significant three-way interaction showed that sequence effects depended jointly on conflict and stimulus complexity. In repeated trials, conflict slowed responses for simple stimuli but showed the opposite pattern for compound stimuli. In switched trials, conflict slowed responses for both simple and compound

stimuli. Complexity increased response times in all conditions, but this effect was largest in congruent-repeated trials and smaller in conflict-repeated and switched trials.

S1.3. Confidence Judgments

Finally, we examined whether confidence ratings in the modified Stroop task are modulated by conflict, stimulus complexity, and sequence. Confidence ratings were analyzed using a linear mixed-effects model with Conflict, Complexity, Sequence, all two-way interactions, and the three-way interaction as fixed effects, with random intercepts for participants. The model was fitted to 26,787 observations from 66 participants. The model yielded a marginal R^2 of .003 and a conditional R^2 of .23. Variability in baseline confidence ratings across participants was reflected in the random-intercept estimates, variance = 0.36, SD = 0.60, ICC = .23.

The omnibus tests revealed a significant main effect of Conflict, $F(1, 26714.21) = 19.85$, $p < .001$. Participants reported lower confidence in conflict trials, M = 12.50, SE = 0.07, 95% CI [12.35, 12.64], than in congruent trials, M = 12.56, SE = 0.07, 95% CI [12.41, 12.70], b = -0.06, SE = 0.01, 95% CI [-0.09, -0.03], $t(26714.21) = -4.46$, $p < .001$. Thus, conflict was associated with a small decrease in subjective confidence.

The main effect of Sequence was significant, $F(1, 26714.75) = 4.37$, $p = .037$. When averaged across Complexity, confidence was lower in switched trials, M = 12.51, SE = 0.07, 95% CI [12.37, 12.66], than in repeated trials, M = 12.54, SE = 0.07, 95% CI [12.39, 12.69], b = −0.03, SE = 0.01, 95% CI [−0.06, −0.00], $t(26714.75) = −2.09$, $p = .037$. The main effect of Complexity was also significant, $F(1, 26714.81) = 11.65$, $p < .001$. When averaged across Sequence, confidence was lower in compound trials, M = 12.50, SE = 0.07, 95% CI [12.36, 12.65], than in simple trials, M = 12.55, SE = 0.07, 95% CI [12.40, 12.70], b = −0.05, SE = 0.01, 95% CI [−0.07, −0.02], $t(26714.81) = −3.41$, $p < .001$. Importantly, however, both marginal effects were qualified by the Sequence × Complexity interaction reported below.

The model revealed a significant Sequence × Complexity interaction, $F(1, 26714.11) = 65.14$, $p < .001$. The sequence effect

was significant for both simple and compound trials, but it differed in direction and was larger for simple trials. For simple trials, participants reported higher confidence in simple-repeated trials, that is, simple trials following simple trials, M = 12.62, SE = 0.08, 95% CI [12.47, 12.77], than in simple-switched trials, that is, simple trials following compound trials, M = 12.48, SE = 0.08, 95% CI [12.33, 12.63], difference = 0.14, SE = 0.02, t = 7.29, p < .001. For compound trials, the sequence effect was reversed: participants reported higher confidence in compound-switched trials, that is, compound trials following simple trials, M = 12.54, SE = 0.08, 95% CI [12.40, 12.69], than in compound-repeated trials, that is, compound trials following compound trials, M = 12.46, SE = 0.08, 95% CI [12.31, 12.61], difference = 0.08, SE = 0.02, t = 4.17, p < .001. Thus, the effect of Sequence reversed across levels of Complexity: confidence was higher in simple-repeated than in simple-switched trials, whereas it was higher in compound-switched than in compound-repeated trials. The Conflict × Sequence × Complexity interaction was not significant, F(1, 26714.64) = 0.02, p = .901. The Conflict × Sequence interaction was not significant, F(1, 26714.50) = 0.02, p = .887, and neither was the Conflict × Complexity interaction, F(1, 26714.08) = 0.10, p = .755.

In summary, confidence was lower in conflict than congruent trials, although this difference was small in absolute terms. Sequence and Complexity also affected confidence, but their marginal effects were qualified by a crossover Sequence × Complexity interaction. Specifically, confidence was higher in simple-repeated than simple-switched trials, whereas the reverse pattern was observed for compound trials.

Table S1. Model-estimated confidence in response correctness as a function of Conflict, Complexity, and Sequence in Experiment 1.

					95% Confidence Intervals	
**Conflict**	**Complexity**	**Sequence**	**Mean**	**SE**	**Lower**	**Upper**

**congruent**	**simple**	**repeat**	12.65	0.08	12.50	12.80
**congruent**	**simple**	**switch**	12.51	0.08	12.36	12.66
**congruent**	**compound**	**repeat**	12.49	0.08	12.34	12.64
**congruent**	**compound**	**switch**	12.57	0.08	12.42	12.72
**conflict**	**simple**	**repeat**	12.59	0.08	12.44	12.74
**conflict**	**simple**	**switch**	12.45	0.08	12.30	12.60
**conflict**	**compound**	**repeat**	12.43	0.08	12.28	12.58
**conflict**	**compound**	**switch**	12.52	0.08	12.37	12.67

S1.4. Exploratory Signal Detection Analyses

We next examined first-order sensitivity and the sensitivity and efficiency of the corresponding confidence judgments across Conflict and Complexity conditions. First-order sensitivity was indexed by *d′*, metacognitive sensitivity by meta-*d′*, and metacognitive efficiency by M-ratio. Estimates were obtained separately for the four Conflict × Complexity conditions.

We first examined first-order task sensitivity. The analysis of *d′* revealed a significant main effect of Conflict, $F(1, 65) = 85.37$, $p < .001$, $\eta p^2 = .57$. First-order sensitivity was higher in congruent than in conflict trials, mean difference = 0.41, $SE = 0.04$, $t(65) = 9.24$, $p < .001$. A significant main effect of Complexity was also observed, $F(1, 65) = 41.59$, $p < .001$, $\eta p^2 = .39$. First-order sensitivity was higher in simple than in compound trials, mean

difference = 0.50, *SE* = 0.08, $t(65) = 6.45$, $p < .001$. We did not detect a Conflict × Complexity interaction, $F(1, 65) = 0.03$, $p = .868$, $\eta p^2 < .01$. Estimated marginal means were 3.70 (*SE* = 0.07, 95% CI [3.56, 3.83]) for simple-congruent trials, 3.28 (*SE* = 0.08, 95% CI [3.11, 3.44]) for simple-conflict trials, 3.19 (*SE* = 0.09, 95% CI [3.02, 3.36]) for compound-congruent trials, and 2.78 (*SE* = 0.11, 95% CI [2.57, 2.99]) for compound-conflict trials. Thus, in the primary analysis, first-order sensitivity was lower under conflict and in compound trials.

The analysis of meta-d′ revealed a significant main effect of complexity, $F(1, 65) = 22.71$, $p < .001$, $\eta p^2 = .26$. Metacognitive sensitivity was higher in simple than compound trials, mean difference = 0.51, SE = 0.11, $t(65) = 4.77$, $p < .001$. The main effect of conflict was not significant, $F(1, 65) = 2.42$, $p = .125$, $\eta p^2 = .04$. However, we observed a significant Complexity × Conflict interaction, $F(1, 65) = 4.54$, $p = .037$, $\eta p^2 = .07$. Follow-up comparisons were therefore used to characterize this interaction. The conflict effect was not detected in simple trials, mean difference = -0.06, SE = 0.09, $t(65) = -0.66$, $p = .509$, Tukey-adjusted $p = .910$. In compound trials, meta-d′ was lower in conflict than in congruent trials before adjustment, mean difference = 0.30, SE = 0.13, $t(65) = 2.24$, $p = .029$; however, the Tukey-adjusted comparison was not significant, $p = .125$. The interaction was more clearly reflected in the complexity effect within each conflict condition. In congruent trials, meta-d′ was higher in simple than in compound trials before adjustment, mean difference = 0.33, SE = 0.14, $t(65) = 2.42$, $p = .018$; however, the Tukey-adjusted comparison was not significant, $p = .083$. In conflict trials, however, meta-d′ was substantially higher in simple than compound trials, mean difference = 0.69, SE = 0.14, $t(65) = 5.08$, $p < .001$, Tukey-adjusted $p < .001$. The significant interaction therefore indicates that the reduction in metacognitive sensitivity from simple to compound trials was strongest when conflict was present.

Table S2. Estimated marginal means of metacognitive sensitivity as a function of Conflict and Complexity in Experiment 1.

				95% Confidence Interval	

Conflict	Complexity	Mean	SE	Lower	Upper
**congruent**	**simple**	4.31	0.10	4.10	4.51
	**compound**	3.97	0.16	3.66	4.29
**conflict**	**simple**	4.37	0.11	4.14	4.59
	**compound**	3.67	0.15	3.37	3.97

We next quantified metacognitive efficiency with the M-ratio, which adjusts meta-d′ for primary-task sensitivity d′ and indexes how well confidence ratings separate correct from incorrect responses (Fleming & Lau, 2014; Maniscalco & Lau, 2012). The 2 × 2 repeated-measures ANOVA revealed a significant main effect of conflict, $F(1, 65) = 15.54$, $p < .001$, $\eta p^2 = .19$. M-ratio values were higher in conflict trials than in congruent trials (mean difference = -0.18, SE = 0.05, $t(65) = -3.94$, $p < .001$). The main effect of complexity was not significant, $F(1, 65) = 0.28$, $p = .597$. The Complexity × Conflict interaction was also not significant, $F(1, 65) = 0.00$, $p = .971$. M-ratio was therefore higher under conflict than under congruent conditions in the primary analysis.

To determine whether the SDT findings depended on the first-order coding used in the primary analysis, we repeated the same analyses after excluding trials with an incorrect line-position response. Under this restriction, complete-sequence correctness was determined entirely by the colour response, such that the binary first-order response corresponded directly to the observed left- or right-hand colour choice on every included trial.

We first examined first-order sensitivity. Conflict continued to reduce d', $F(1, 65) = 91.68$, $p < .001$, $\eta p^2 = .59$, with higher sensitivity in congruent than conflict trials, mean difference = 0.44, $SE = 0.05$, $t(65) = 9.57$, $p < .001$. In contrast, after trials with an incorrect line-position response were excluded, we no longer detected an effect of Complexity, $F(1, 65) = 3.31$, $p = .073$, $\eta p^2 = .05$, or a Conflict × Complexity interaction, $F(1, 65) = 0.64$, $p = .426$, $\eta p^2 = .01$. Estimated marginal means were 4.03 for simple-congruent, 3.57 for simple-conflict, 4.07 for compound-congruent, and 3.66 for compound-conflict trials. Thus, Conflict reliably reduced first-order sensitivity, whereas the Complexity-related reduction observed in the primary analysis disappeared, as expected once errors in the additional line-position response no longer contributed to first-order correctness.

Metacognitive sensitivity showed a different pattern. Complexity continued to reduce meta-d', $F(1, 65) = 25.47$, $p < .001$, $\eta p^2 = .28$, whereas the main effect of Conflict was not significant, $F(1, 65) = 1.15$, $p = .288$, $\eta p^2 = .02$. Importantly, the Conflict × Complexity interaction was again significant, $F(1, 65) = 9.46$, $p = .003$, $\eta p^2 = .13$. Estimated marginal means were 4.28 for simple-congruent, 4.39 for simple-conflict, 4.04 for compound-congruent, and 3.77 for compound-conflict trials. Follow-up comparisons showed that the simple–compound difference was significant under conflict, mean difference = 0.61, $SE = 0.12$, $t(65) = 5.29$, $p < .001$, Tukey-adjusted $p < .001$. In congruent trials, the corresponding difference did not reach significance after correction, mean difference = 0.24, $SE = 0.09$, $t(65) = 2.62$, $p = .011$, Tukey-adjusted $p = .052$. The interaction therefore reflected a particularly pronounced reduction in metacognitive sensitivity in compound trials under conflict. Thus, unlike the Complexity effect on d', the Complexity-related reduction in meta-d' remained when first-order responses corresponded directly to the observed colour response and the additional line-position response was correct.

Finally, we examined metacognitive efficiency. The main effect of Conflict observed in the primary analysis was retained after trials with an incorrect line-position response were excluded, $F(1, 65) = 22.77$, $p < .001$, $\eta p^2 = .26$. Thus, M-ratio remained higher under conflict when the binary first-order response corresponded directly to the participant's observed

colour response on every included trial. In the sensitivity analysis, however, this effect was qualified by Complexity. We observed a significant main effect of Complexity, $F(1, 65) = 26.66$, $p < .001$, $\eta p^2 = .29$, and a significant Conflict × Complexity interaction, $F(1, 65) = 9.74$, $p = .003$, $\eta p^2 = .13$. Estimated marginal means were 1.07 for simple-congruent, 1.25 for simple-conflict, 0.99 for compound-congruent, and 1.04 for compound-conflict trials. In simple trials, M-ratio was higher under conflict than under congruent conditions, mean difference = 0.19, $SE = 0.04$, $t(65) = 5.17$, $p < .001$, Tukey-adjusted $p < .001$, whereas the conflict-related difference was not significant in compound trials, mean difference = 0.05, $SE = 0.03$, $t(65) = 1.61$, $p = .113$, Tukey-adjusted $p = .383$. M-ratio was also lower in compound than simple trials under congruent conditions, mean difference = 0.08, $SE = 0.03$, $t(65) = 3.00$, $p = .004$, Tukey-adjusted $p = .019$, and under conflict, mean difference = 0.21, $SE = 0.04$, $t(65) = 4.90$, $p < .001$, Tukey-adjusted $p < .001$. The conflict-related increase in M-ratio was therefore evident in simple trials, whereas no reliable conflict-related difference was detected in compound trials. M-ratio was also lower in compound than simple trials under both levels of Conflict, with a larger difference under conflict.

Taken together, after trials with an incorrect line-position response had been excluded, Conflict continued to lower first-order sensitivity: d′ remained lower in conflict than in congruent trials. In contrast, d' no longer differed significantly between simple and compound trials, and there was no Conflict × Complexity interaction. Metacognitive sensitivity showed a different pattern. Meta-d' was lower in compound than in simple trials, whereas there was no overall difference between conflict and congruent trials. Importantly, the effect of Complexity depended on Conflict: the decrease in meta-d' from simple to compound trials was larger under conflict than under congruent conditions. For metacognitive efficiency, M-ratio was overall higher in conflict than in congruent trials and lower in compound than in simple trials. These effects interacted: the conflict-related increase in M-ratio was present in simple trials but not in compound trials, and the decrease in M-ratio from simple to compound trials was larger under conflict. Thus, excluding trials with an incorrect line-position response

preserved the conflict-related reduction in d', the complexity-related reduction in meta-d' and its stronger expression under conflict, and the overall increase in M-ratio under conflict. At the same time, the Complexity effect on d' was no longer detected, while M-ratio additionally showed a Complexity effect and a Conflict × Complexity interaction.

Table S3. Estimated marginal means of metacognitive efficiency as a function of Conflict and Complexity in Experiment 1.

				95% Confidence Interval	
**Conflict**	**Complexity**	**Mean**	**SE**	**Lower**	**Upper**
**congruent**	**simple**	1.19	0.03	1.12	1.25
	**compound**	1.21	0.07	1.07	1.36
**conflict**	**simple**	1.37	0.04	1.29	1.45
	**compound**	1.39	0.06	1.28	1.51

S2. Supplementary Results: Experiment 2

S2.1. Accuracy

Firstly, we examined the influence of conflict, stimulus complexity, and sequence on response accuracy in the Stroop test. For this purpose we fitted a generalized linear mixed-effects model with a binomial distribution and a logit link function. The model included Conflict, Complexity, Sequence, and their

interactions as fixed effects, and a random intercept of a participant. The model converged successfully and was fitted to 21,049 trials from 47 participants. The fixed-effects structure was significant, marginal $R^2 = .02$, $\chi^2(7) = 174.82$, $p < .001$. Conditional R^2 was .24 after including participant-level variability. The random intercept variance was 0.97, SD = 0.99, ICC = .23.

The model revealed a significant main effect of Conflict, $\chi^2(1) = 163.91$, $p < .001$. Participants responded more accurately in congruent trials, M = .89, SE = .01, 95% CI [.86, .92], than in conflict trials, M = .83, SE = .02, 95% CI [.79, .87], OR = 1.66, SE = 0.04, 95% CI [1.53, 1.79], $z = 12.80$, $p < .001$. Thus, conflict between task-relevant and task-irrelevant information reduced overt response accuracy.

The main effects of Complexity and Sequence were not significant. We did not detect differences in response accuracy between simple and compound trials, $\chi^2(1) = 0.00$, $p = .969$, OR = 1.00, SE = 0.04, 95% CI [0.92, 1.08], $z = -0.04$, $p = .969$. We also did not detect differences in accuracy between switched and repeated trials, $\chi^2(1) = 0.81$, $p = .367$, OR = 0.97, SE = 0.04, 95% CI [0.89, 1.04], $z = -0.90$, $p = .367$. The Conflict × Complexity interaction was not significant, $\chi^2(1) = 0.59$, $p = .442$, and neither was the Conflict × Sequence interaction, $\chi^2(1) = 0.12$, $p = .726$.

The Complexity × Sequence interaction was significant, $\chi^2(1) = 3.96$, $p = .047$. However, this two-way interaction was qualified by a significant Conflict × Complexity × Sequence interaction, $\chi^2(1) = 5.40$, $p = .020$. We therefore decomposed the three-way interaction by examining the conflict effect separately within each combination of Complexity and Sequence.

The conflict effect was significant in all four Complexity × Sequence conditions, but its magnitude varied across conditions. In compound-repeated trials, that is, compound trials following compound trials, participants were more accurate in congruent than conflict trials, OR = 1.58, SE = 0.08, 95% CI [1.36, 1.84], $z = 5.91$, $p < .001$. In simple-repeated trials, that is, simple trials following simple trials, the conflict effect was also significant, OR = 1.79, SE = 0.08, 95% CI [1.53, 2.08], $z = 7.38$, $p < .001$. The Conflict × Complexity interaction was not significant within repeated trials, $\chi^2(1) = 1.23$, $p = .267$, indicating that the difference between the conflict effect in simple-repeated and compound-repeated trials was not reliable.

In switched trials, the conflict effect was again significant for both levels of Complexity. In compound-switched trials, that is, compound trials following simple trials, participants were more accurate in congruent than conflict trials, OR = 1.85, SE = 0.08, 95% CI [1.58, 2.16], z = 7.61, $p < .001$. In simple-switched trials, that is, simple trials following compound trials, the conflict effect was also significant, OR = 1.45, SE = 0.08, 95% CI [1.24, 1.69], z = 4.72, $p < .001$. In contrast to repeated trials, the Conflict × Complexity interaction was significant within switched trials, $\chi^2(1) = 4.70$, p = .030, indicating that the conflict effect was larger in compound-switched than in simple-switched trials.

In summary, response accuracy was lower in conflict than congruent trials, confirming a robust interference cost in Experiment 2. We did not detect main effects of Complexity and Sequence on accuracy. However, the significant three-way interaction showed that the magnitude of the conflict effect depended on the combination of Complexity and Sequence. The conflict effect was detected in all conditions, but it was especially pronounced in compound-switched and simple-repeated trials. Thus, Experiment 2 reproduced the expected behavioral cost of Conflict, whereas the effects of Complexity and Sequence on overt accuracy were expressed mainly through their modulation of the conflict effect.

Table S4. Model-estimated response accuracy across Conflict, Complexity, and Sequence conditions in Experiment 2.

					95% Confidence Intervals	
**Conflict**	**Complexity**	**Sequence**	**Mean**	**SE**	**Lower**	**Upper**
**congruent**	**simple**	**repeat**	0.90	0.01	0.87	0.92
**congruent**	**simple**	**switch**	0.88	0.02	0.84	0.91
**congruent**	**compound**	**repeat**	0.89	0.02	0.85	0.91

**congruent**	**compound**	**switch**	0.90	0.01	0.87	0.92
**conflict**	**simple**	**repeat**	0.84	0.02	0.79	0.87
**conflict**	**simple**	**switch**	0.83	0.02	0.79	0.87
**conflict**	**compound**	**repeat**	0.83	0.02	0.79	0.87
**conflict**	**compound**	**switch**	0.83	0.02	0.78	0.87

S2.2. Partial-Error Occurrence

We next examined whether the experimental conditions affected partial-error occurrence in fully correct trials. The analysis was restricted to eligible trials in which both the colour response and the subsequent line-position response were correct and for which a valid EMG classification was available. Within this set, partial-error occurrence was coded as present when a verified incorrect-hand activation preceded the subsequent correct-hand activation and as absent otherwise. A binomial generalized linear mixed-effects model included Conflict, Complexity, Sequence, and their interactions as fixed effects and a random intercept for participant. The model converged successfully and was fitted to 17,413 trials from 47 participants. The fixed-effects structure was significant, $\chi^2(7) = 25.52$, $p < .001$, marginal $R^2 = .004$; conditional $R^2 = .14$. A main effect of Conflict was detected, $\chi^2(1) = 10.92$, $p = .001$. Among trials in which both task responses were correct, partial errors were more likely in conflict trials, M = .108, SE = .011, 95% CI [.089, .131], than in congruent trials, M = .093, SE = .009, 95% CI [.077, .114], OR = 1.18, $z = 3.31$, $p = .001$. A main effect of Complexity was also detected, $\chi^2(1) = 11.81$, $p < .001$. Partial errors were less likely in compound trials, M = .093, SE = .009, 95% CI [.076, .113], than in simple trials,

$M = .109$, $SE = .011$, 95% CI [.089, .132], $OR = 0.84$, $z = -3.44$, $p < .001$. We did not detect a main effect of Sequence, $\chi^2(1) = 0.49$, $p = .482$, or any interactions, all $ps \geq .358$. Thus, among trials in which both the colour and line-position responses were correct, partial errors occurred more frequently under conflict and less frequently in compound than in simple trials. We did not detect sequence-related differences in partial-error occurrence.

S2.3. Response Times

We then examined whether conflict, stimulus complexity, and sequence affected the latency of correct overt responses. Response times were analysed to determine whether Experiment 2 reproduced the costs of cognitive control observed in Experiment 1 and in previous work using related control manipulations (Rietbergen et al., [39]). The analysis was performed on fully correct trials only. We fitted a linear mixed-effects model with Conflict, Complexity, Sequence, all two-way interactions, and the three-way interaction as fixed effects. The model also included a random intercept for participant. The model was estimated on 17,413 fully correct trials from 47 participants.

The model converged successfully. The fixed-effects structure accounted for a significant portion of response-time variability, marginal $R^2 = .01$, $\chi^2(7) = 375.04$, $p < .001$. Conditional R^2 was substantially higher, conditional $R^2 = .46$, indicating that response times were strongly shaped by stable between-participant differences. This was further supported by the random-effect structure, which showed considerable participant-level variability in baseline response speed, variance = 18046, $SD = 134.33$, $ICC = .46$.

The model revealed a significant main effect of Conflict, $F(1, 17361) = 286.02$, $p < .001$. Response times were longer in conflict trials ($M = 552.4$ ms, $SE = 19.66$, 95% CI [512.8, 592.0]) than in congruent trials ($M = 514.5$ ms, $SE = 19.66$, 95% CI [474.9, 554.0]). The estimated interference cost was 37.95 ms, $SE = 2.24$, 95% CI [33.56, 42.34], $t(17361) = 16.91$, $p < .001$. Thus, as in Experiment 1, conflict between task-relevant and task-irrelevant information

reliably delayed responding, indicating increased demands on interference control.

The model also revealed a significant main effect of Complexity, $F(1, 17359) = 78.20$, $p < .001$. Response times were longer in compound trials ($M = 543.3$ ms, $SE = 19.66$, 95% CI [503.7, 582.9]) than in simple trials ($M = 523.6$ ms, $SE = 19.66$, 95% CI [484.0, 563.1]). The estimated complexity cost was 19.71 ms, $SE = 2.23$, 95% CI [15.34, 24.08], $t(17359) = 8.84$, $p < .001$. Consistent with the logic of Rietbergen et al. [39], increasing stimulus–response complexity prolonged response times, suggesting greater demands on updating-like processing and response selection.

The main effect of Sequence did not reach significance, $F(1, 17359) = 3.61$, $p = .057$. Although switch trials were numerically slower ($M = 535.6$ ms, $SE = 19.66$) than repeat trials ($M = 531.3$ ms, $SE = 19.66$), the estimated switch cost was small and its confidence interval included zero, $b = 4.24$ ms, $SE = 2.23$, 95% CI [−0.13, 8.61], $t(17359) = 1.90$, $p = .057$. Thus, unlike the robust interference and complexity costs, sequence-related slowing was weak in Experiment 2.

The Conflict × Complexity interaction did not reach significance, $F(1, 17359) = 3.40$, $p = .065$. No other interactions reached significance. The Conflict × Sequence interaction was not significant, $F(1, 17359) = 2.80$, $p = .094$. The Complexity × Sequence interaction was also not significant, $F(1, 17359) = 0.33$, $p = .569$, and neither was the Conflict × Complexity × Sequence interaction, $F(1, 17359) = 0.06$, $p = .802$.

Taken together, the response-time results replicated main costs of conflict and stimulus–response complexity from Experiment 1. Response times increased when irrelevant and relevant stimulus dimensions were not congruent and when the task involved compound rather than simple stimulus–response processing. In contrast, sequence-related slowing was weak. No interaction reached significance.

Table S5. Model-estimated response times across task conditions in Experiment 2. Estimated marginal means are expressed in milliseconds. Confidence intervals represent 95% confidence intervals.

						95% Confidence Intervals	
**Conflict**	**Complexity**	**Sequence**	**Mean**	**SE**	**df**	**Lower**	**Upper**
**congruent**	**simple**	**repeat**	498.2	19.82	47.84	458.3	538.1
**congruent**	**simple**	**switch**	506.9	19.85	48.07	467.0	546.8
**congruent**	**compound**	**repeat**	522.7	19.83	47.93	482.9	562.6
**congruent**	**compound**	**switch**	530.0	19.84	48.01	490.1	569.9
**conflict**	**simple**	**repeat**	543.4	19.85	48.12	503.5	583.4
**conflict**	**simple**	**switch**	545.8	19.87	48.24	505.8	585.7
**conflict**	**compound**	**repeat**	560.9	19.86	48.17	520.9	600.8
**conflict**	**compound**	**switch**	559.5	19.87	48.28	519.6	599.5

S2.4. Premotor Time

To determine whether the observed response-time effects emerged before or after the onset of measurable muscle activation, we decomposed response time into premotor and motor components. Premotor time was defined as the interval between stimulus onset and the onset of the correct-hand EMG activation, whereas motor time was defined as the interval between the onset of the correct-hand EMG activation and the overt button press. Both analyses were restricted to fully correct

pure-correct trials with valid EMG onset and response markers. Two trials identified as outliers in the premotor-time box plot were excluded from the analysis.

Premotor time was analyzed for pure-correct trials using a linear mixed-effects model. The model included Conflict, Complexity, Sequence, and their interactions as fixed effects, with a random participant-specific intercept. The model was estimated on 13,788 trials from 47 participants.

Descriptively, the mean premotor time was 411.40 ms (*SD* = 176.38), with a median of 376.46 ms. The fixed-effects structure significantly accounted for variability in premotor time, marginal $R^2 = .01$, $\chi^2(7) = 282.64$, $p < .001$. The conditional R^2 was .46, indicating that participant-level variability accounted for a substantial proportion of variance in premotor time. The random intercept for a participant showed considerable between-participant variability, ICC = .46.

The model revealed a significant main effect of Conflict, $F(1, 13736.85) = 199.60$, $p < .001$. Premotor times were longer on conflict trials (*M* = 425.38 ms, *SE* = 17.98, 95% CI [389.19, 461.56]) than on congruent trials (*M* = 392.86 ms, *SE* = 17.97, 95% CI [356.70, 429.02]). The estimated conflict effect was 32.52 ms, *SE* = 2.30, 95% CI [28.00, 37.03], $t(13736.85) = 14.13$, $p < .001$.

There was also a significant main effect of Complexity, $F(1, 13734.16) = 73.32$, $p < .001$. Premotor times were longer on compound trials (*M* = 418.89 ms, *SE* = 17.97, 95% CI [382.72, 455.06]) than on simple trials (*M* = 399.35 ms, *SE* = 17.97, 95% CI [363.18, 435.52]). The estimated complexity effect was 19.54 ms, *SE* = 2.28, 95% CI [15.07, 24.02], $t(13734.16) = 8.56$, $p < .001$.

The main effect of Sequence was not significant, $F(1, 13734.24) = 1.10$, $p = .295$. Premotor times were similar on switch trials (*M* = 410.31 ms, *SE* = 17.98, 95% CI [374.14, 446.49]) and repeat trials (*M* = 407.92 ms, *SE* = 17.97, 95% CI [371.76, 444.09]). The estimated sequence effect was 2.39 ms, *SE* = 2.28, 95% CI [-2.09, 6.87], $t(13734.24) = 1.05$, $p = .295$.

None of the interactions reached significance. The Conflict × Complexity interaction was not significant, $F(1, 13734.26) = 1.51$, $p = .220$. The Conflict × Sequence interaction was not significant, $F(1, 13734.24) = 2.17$, $p = .141$, nor was the Complexity × Sequence interaction, $F(1, 13734.17) = 1.24$, $p = .265$. The three-way Conflict

× Complexity × Sequence interaction was also not significant, $F(1, 13734.28) = 1.48$, $p = .223$.

Taken together, premotor times were longer in conflict than in congruent trials and longer in compound than in simple trials. We did not detect an effect of Sequence or any interactions among Conflict, Complexity, and Sequence.

S2.5. Motor Time

Motor times outside the range defined by the overall median ±3 *SD* (median = 92.77 ms, *SD* = 34.12 ms) were excluded. Motor time was analyzed using a linear mixed-effects model with Conflict, Complexity, Sequence, and their interactions as fixed effects, and a random intercept for participant. The model was estimated on 13,501 trials from 47 participants.

The fixed-effects structure significantly accounted for variability in motor time, marginal $R^2 = .002$, $\chi^2(7) = 47.15$, $p < .001$. The conditional R^2 was .56, indicating substantial participant-level variability in motor time. Consistent with this, the random intercept for participant accounted for a large proportion of variance, ICC = .56.

The model revealed a significant main effect of Conflict, $F(1, 13448.87) = 11.19$, $p < .001$. Motor time was longer on conflict than on congruent trials, $b = 1.17$ ms, $SE = 0.35$, 95% CI [0.48, 1.85], $t(13448.87) = 3.35$, $p < .001$.

There was also a significant main effect of Complexity, $F(1, 13447.10) = 30.92$, $p < .001$. Motor time was longer on compound than on simple trials, $b = 1.93$ ms, $SE = 0.35$, 95% CI [1.25, 2.60], $t(13447.10) = 5.56$, $p < .001$.

The main effect of Sequence was not significant, $F(1, 13447.14) = 0.00$, $p = .959$. Motor time did not differ between switch and repeat trials, $b = -0.02$ ms, $SE = 0.35$, 95% CI [-0.70, 0.66], $t(13447.14) = -0.05$, $p = .959$.

No interaction reached significance. The Conflict × Complexity interaction was not significant, $F(1, 13447.16) = 0.09$, $p = .759$. The Conflict × Sequence interaction was not significant, $F(1, 13447.16) = 0.02$, $p = .881$. The Complexity × Sequence interaction did not reach significance, $F(1, 13447.10) = 3.33$, $p = .068$. The three-way Conflict × Complexity × Sequence interaction was also not significant, $F(1, 13447.20) = 0.49$, $p = .483$.

Taken together, motor times were longer in conflict than in congruent trials and longer in compound than in simple trials.

We did not detect an effect of Sequence or any interactions among Conflict, Complexity, and Sequence.

S2.6. Partial-Error Confidence

The central analysis examined whether confidence in partial-error occurrence was related to EMG-derived characteristics of the incorrect motor activation and whether these relationships varied across task conditions.

To address this question, we fitted a cumulative link mixed model (CLMM) with confidence ratings as the ordinal dependent variable. The model included incorrect-burst EMG surface, correction time, Conflict, Complexity, and Sequence as fixed effects, together with all two-way interactions among task factors, interactions of incorrect-burst surface and correction time with each task factor, and the Conflict × Complexity × Sequence interaction. A random intercept for participants was included. The model was specified as:

Confidence ~ Burst_surface + Correction_time + Conflict * Complexity * Sequence + Burst_surface:Conflict + Burst_surface:Complexity + Burst_surface:Sequence + Correction_time:Conflict + Correction_time:Complexity + Correction_time:Sequence + (1 | participant).

Categorical task factors in both CLMMs were coded using GAMLj simple contrasts.

Incorrect-burst surface and correction time were log10-transformed to reduce positive skew and subsequently z-standardised separately within each participant. Response time (RT) was processed in the same manner before its inclusion in the RT-adjusted model. Consequently, coefficients for the continuous predictors represented the change in the log-odds of reporting a higher partial-error confidence category associated with a one-participant-specific-standard-deviation increase in the corresponding log10-transformed predictor.

The model converged successfully and was estimated on 1,689 partial-error trials from 44 participants. The fixed-effects structure was significant, $\chi^2(15) = 187.39$, $p < .001$, with a marginal R^2 of .08 and a conditional R^2 of .41. The participant-level random intercept accounted for substantial between-

participant variability in confidence, variance = 1.89, SD = 1.37, ICC = .36.

Incorrect EMG surface was a strong positive predictor of confidence. Larger incorrect EMG surface was associated with higher confidence that a partial error had occurred, $b = 0.50$, $SE = 0.05$, $OR = 1.64$, 95% CI [1.50, 1.80], $z = 10.47$, $p < .001$. Model-estimated confidence increased from 2.40 at −1 SD of incorrect EMG surface to 2.80 at the mean and 3.24 at +1 SD. Thus, confidence increased as the incorrect motor activation became more pronounced.

Correction time also positively predicted confidence, $b = 0.30$, $SE = 0.05$, $OR = 1.35$, 95% CI [1.23, 1.48], $z = 6.16$, $p < .001$. Model-estimated confidence increased from 2.56 at −1 SD of correction time to 2.80 at the mean and 3.06 at +1 SD. Thus, confidence was related both to the incorrect EMG activation and to the time separating the initial incorrect activation from the subsequent correct activation.

Partial-error confidence was lower in simple than in compound trials, with model-estimated confidence of 2.66 and 2.94, respectively. This difference was statistically significant, $b = -0.32$, $SE = 0.09$, $OR = 0.73$, 95% CI [0.61, 0.87], $z = -3.53$, $p < .001$. In contrast, neither Conflict, $b = -0.14$, $SE = 0.09$, $OR = 0.87$, 95% CI [0.73, 1.05], $z = -1.48$, $p = .140$, nor Sequence, $b = -0.03$, $SE = 0.09$, $OR = 0.97$, 95% CI [0.81, 1.16], $z = -0.30$, $p = .767$, significantly predicted confidence.

Importantly, the association between incorrect EMG surface and confidence was moderated by Complexity, $b = 0.24$, $SE = 0.09$, $OR = 1.27$, 95% CI [1.06, 1.52], $z = 2.54$, $p = .011$. Confidence increased with incorrect EMG surface under both levels of Complexity, but the increase was larger in simple trials. In simple trials, model-estimated confidence increased from 2.19 at −1 SD of incorrect EMG surface to 3.20 at +1 SD; in compound trials, the corresponding increase was from 2.62 to 3.28. Thus, incorrect EMG surface was related to confidence in both conditions, but more strongly during simple than compound stimulus–response processing.

Complexity also moderated the effect of correction time, $b = 0.27$, $SE = 0.10$, $OR = 1.31$, 95% CI [1.08, 1.59], $z = 2.78$, $p = .005$. Simple-effects analyses showed that correction time positively predicted confidence in compound trials, $b = 0.16$, $SE = 0.06$, OR

= 1.18, 95% CI [1.04, 1.34], $z = 2.54$, $p = .011$, and in simple trials, $b = 0.43$, $SE = 0.07$, $OR = 1.54$, 95% CI [1.34, 1.78], $z = 5.98$, $p < .001$. The association was therefore reliable in both conditions but substantially stronger in simple trials. No other interaction reached statistical significance (all $ps \geq .624$).

We next fitted an additional model including RT as a covariate to test whether the associations between EMG-derived response-process characteristics and confidence could be accounted for by global response slowing. The RT-adjusted model was specified as:

Confidence ~ Burst_surface + Correction_time + RT + Conflict * Complexity * Sequence + Burst_surface:Conflict + Burst_surface:Complexity + Burst_surface:Sequence + Correction_time:Conflict + Correction_time:Complexity + Correction_time:Sequence + (1 | participant).

The RT-adjusted model showed better fit than the model without RT, with AIC = 5282.87 compared with AIC = 5332.20 for the model without RT, and explained slightly more variance, marginal $R^2 = .09$, conditional $R^2 = .43$. The RT-adjusted fixed-effects structure was significant, $\chi^2(16) = 238.72$, $p < .001$. RT was itself a significant positive predictor of confidence, $b = 0.37$, $SE = 0.05$, $OR = 1.45$, 95% CI [1.32, 1.61], $z = 7.14$, $p < .001$, indicating that slower responses were associated with higher confidence that a partial error had occurred.

Crucially, incorrect EMG surface remained a robust predictor after controlling for RT, $b = 0.45$, $SE = 0.05$, $OR = 1.56$, 95% CI [1.43, 1.72], $z = 9.41$, $p < .001$. Correction time was attenuated but remained significant, $b = 0.15$, $SE = 0.05$, $OR = 1.16$, 95% CI [1.04, 1.28], $z = 2.81$, $p = .005$. Partial-error confidence remained lower in simple than compound trials after adjustment for RT. The Complexity effect remained significant, $b = -0.29$, $SE = 0.09$, $OR = 0.75$, 95% CI [0.63, 0.90], $z = -3.17$, $p = .002$. Conflict, $b = -0.08$, $SE = 0.09$, $OR = 0.93$, 95% CI [0.78, 1.11], $z = -0.82$, $p = .411$, and Sequence, $b = -0.04$, $SE = 0.09$, $OR = 0.96$, 95% CI [0.81, 1.15], $z = -0.40$, $p = .689$, remained nonsignificant.

The Incorrect EMG surface × Complexity interaction also remained significant after controlling for RT, $b = 0.24$, $SE = 0.09$, $OR = 1.27$, 95% CI [1.06, 1.53], $z = 2.58$, $p = .010$. Confidence increased with incorrect EMG surface in both complexity

conditions, but the increase remained larger in simple trials. In simple trials, model-estimated confidence increased from 2.23 at −1 SD of incorrect EMG surface to 3.17 at +1 SD; in compound trials, the corresponding increase was from 2.65 to 3.22. Thus, the stronger correspondence between incorrect EMG surface and confidence in simple trials could not be explained by differences in overall response speed.

The Correction time × Complexity interaction likewise remained significant after controlling for RT, b = 0.24, SE = 0.10, OR = 1.27, 95% CI [1.05, 1.54], z = 2.51, p = .012. However, the simple-effects pattern changed after RT adjustment. Correction time no longer predicted confidence in compound trials, b = 0.03, SE = 0.07, OR = 1.03, 95% CI [0.90, 1.17], z = 0.38, p = .704, whereas the association remained significant in simple trials, b = 0.27, SE = 0.07, OR = 1.31, 95% CI [1.13, 1.51], z = 3.60, $p < .001$. Thus, after accounting for overall response speed, correction time remained associated with partial-error confidence only in simple trials. No other interaction reached statistical significance in the RT-adjusted model (all ps ≥ .675).

Taken together, partial-error confidence was positively associated with incorrect-burst surface and correction time. The association with incorrect-burst surface remained robust after RT adjustment, whereas the correction-time association was attenuated and remained reliable only in simple trials. Stimulus Complexity moderated both relationships, with stronger associations in simple than compound trials. Thus, the relationship between measurable characteristics of the partial-error event and confidence varied with task context.

S2.7. Exploratory Signal Detection Analyses

Finally, we examined initial motor-response sensitivity, metacognitive sensitivity, and metacognitive efficiency of partial-error judgments. Initial motor-response sensitivity was indexed by d′, metacognitive sensitivity by meta-d′, and metacognitive efficiency by M-ratio. Estimates were obtained separately for each Conflict × Complexity condition.

In the binary SDT representation, the stimulus class was the response hand required by the colour stimulus and the response class was the hand of the first detected EMG burst. Consequently, pure-correct and partial-error trials differed in the hand of the initial motor response. In pure-correct trials, the initial response occurred in the stimulus-required hand, whereas in partial-error trials it occurred in the opposite hand and was subsequently corrected before the overt response.

The analysis included 42 participants with available d′ estimates in all four Conflict × Complexity conditions. A 2 × 2 repeated-measures ANOVA revealed a significant main effect of Conflict, $F(1, 41) = 13.88$, $p < .001$, $\eta p^2 = .25$. Initial motor-response sensitivity was higher on congruent than on conflict trials, mean difference = 0.23, $SE = 0.06$, $t(41) = 3.73$, $p < .001$. A significant main effect of Complexity was also observed, $F(1, 41) = 11.47$, $p = .002$, $\eta p^2 = .22$. Initial motor-response sensitivity was higher on compound than on simple trials, mean difference = 0.16, $SE = 0.05$, $t(41) = 3.39$, $p = .002$. There was no evidence of a Conflict × Complexity interaction, $F(1, 41) = 0.41$, $p = .523$, $\eta p^2 = .01$.

Thus, initial motor-response sensitivity was lower on conflict than on congruent trials but higher on compound than on simple trials. Descriptively, this pattern was consistent with the previously reported incidence of partial errors, which was higher on conflict trials but lower on compound trials.

We next examined metacognitive sensitivity, defined here as the extent to which the six graded confidence levels discriminated between pure-correct and partial-error trials. The analysis included 42 participants with estimable meta-d′ values in all four Conflict × Complexity conditions.

A 2 × 2 repeated-measures ANOVA revealed significant main effects of Conflict, $F(1, 41) = 10.70$, $p = .002$, $\eta p^2 = .21$, and Complexity, $F(1, 41) = 8.51$, $p = .006$, $\eta p^2 = .17$. Meta-d′ was higher on congruent than on conflict trials, mean difference = 0.31, $SE = 0.10$, $t(41) = 3.27$, $p = .002$, and higher on simple than on compound trials, mean difference = 0.31, $SE = 0.11$, $t(41) = 2.92$, $p = .006$. There was no evidence of a Conflict × Complexity interaction, $F(1, 41) = 1.34$, $p = .254$, $\eta p^2 = .03$.

Descriptively, the estimated marginal means were highest in the simple-congruent condition and lowest in the compound-conflict condition, with intermediate estimates in the simple-

conflict and compound-congruent conditions. Condition-specific estimated marginal means, standard errors, and 95% confidence intervals are reported in Table S6 and displayed in Figure S1.

Thus, both conflict and stimulus complexity reduced metacognitive sensitivity to partial errors, with no evidence that these variables interacted.

Table S6. Estimated marginal means of metacognitive sensitivity (meta-d′) as a function of Conflict and Complexity in Experiment 2

				95% Confidence Interval	
**Complexity**	**Conflict**	**Mean**	**SE**	**Lower**	**Upper**
**simple**	**congruent**	1.77	0.19	1.39	2.15
	**conflict**	1.35	0.12	1.11	1.59
**compound**	**congruent**	1.35	0.17	1.00	1.70
	**conflict**	1.14	0.16	0.82	1.46

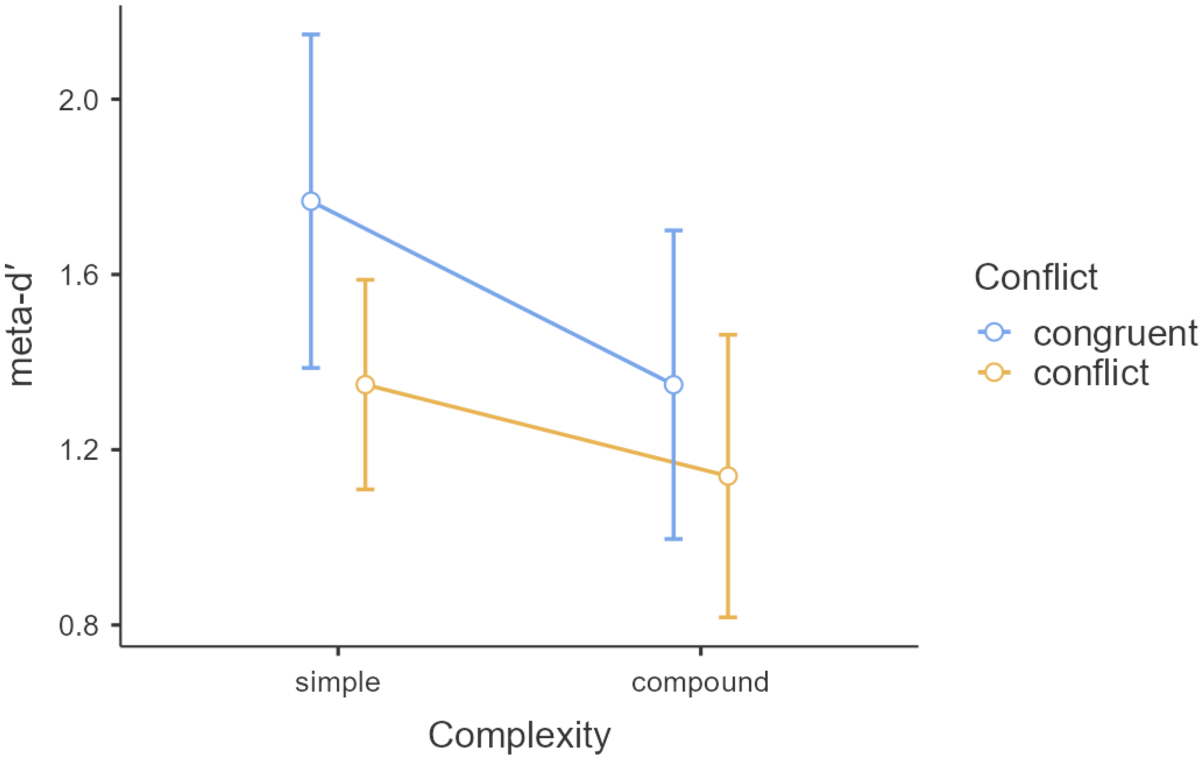

Figure S1. Marginal means of metacognitive sensitivity (meta-d′) as a function of Conflict and Complexity in Experiment 2.

We next examined metacognitive efficiency using M-ratio. The analysis included 42 participants with estimable M-ratio values in all four Conflict × Complexity conditions.

A 2 × 2 repeated-measures ANOVA showed no significant main effect of Conflict, $F(1, 41) = 1.28$, $p = .264$, $\eta p^2 = .03$. In contrast, there was a significant main effect of Complexity, $F(1, 41) = 13.45$, $p < .001$, $\eta p^2 = .25$. M-ratio was higher on simple than on compound trials, mean difference = 0.21, $SE = 0.06$, $t(41) = 3.67$, $p < .001$. There was no evidence of a Conflict × Complexity interaction, $F(1, 41) = 0.03$, $p = .872$, $\eta p^2 < .01$.

Descriptively, the estimated marginal means were higher in both simple conditions than in either compound condition, whereas differences associated with Conflict were comparatively small. Condition-specific estimated marginal

means, standard errors, and 95% confidence intervals are reported in Table S7 and displayed in Figure S2.

Taken together, Conflict and Complexity had different effects on first-order motor and metacognitive measures. Conflict was associated with a higher incidence of partial errors and correspondingly lower d′, as well as lower meta-d′, whereas we did not detect a significant effect of Conflict on M-ratio. Complexity showed a different pattern: partial errors were less frequent and *d′* was higher in compound than simple trials, whereas both meta-*d′* and M-ratio were lower in compound trials. Because partial-error trials were defined by initial motor responses occurring in the hand opposite to that required by the stimulus, the higher d′ observed in compound trials likely reflects the lower incidence of such responses.

Table S7. Estimated marginal means of metacognitive efficiency (M-ratio) as a function of Conflict and Complexity in Experiment 2.

				95% Confidence Interval	
**Complexity**	**Conflict**	**Mean**	**SE**	**Lower**	**Upper**
**simple**	**congruent**	0.78	0.09	0.60	0.96
	**conflict**	0.72	0.09	0.55	0.89
**compound**	**congruent**	0.56	0.07	0.42	0.70
	**conflict**	0.51	0.08	0.36	0.67

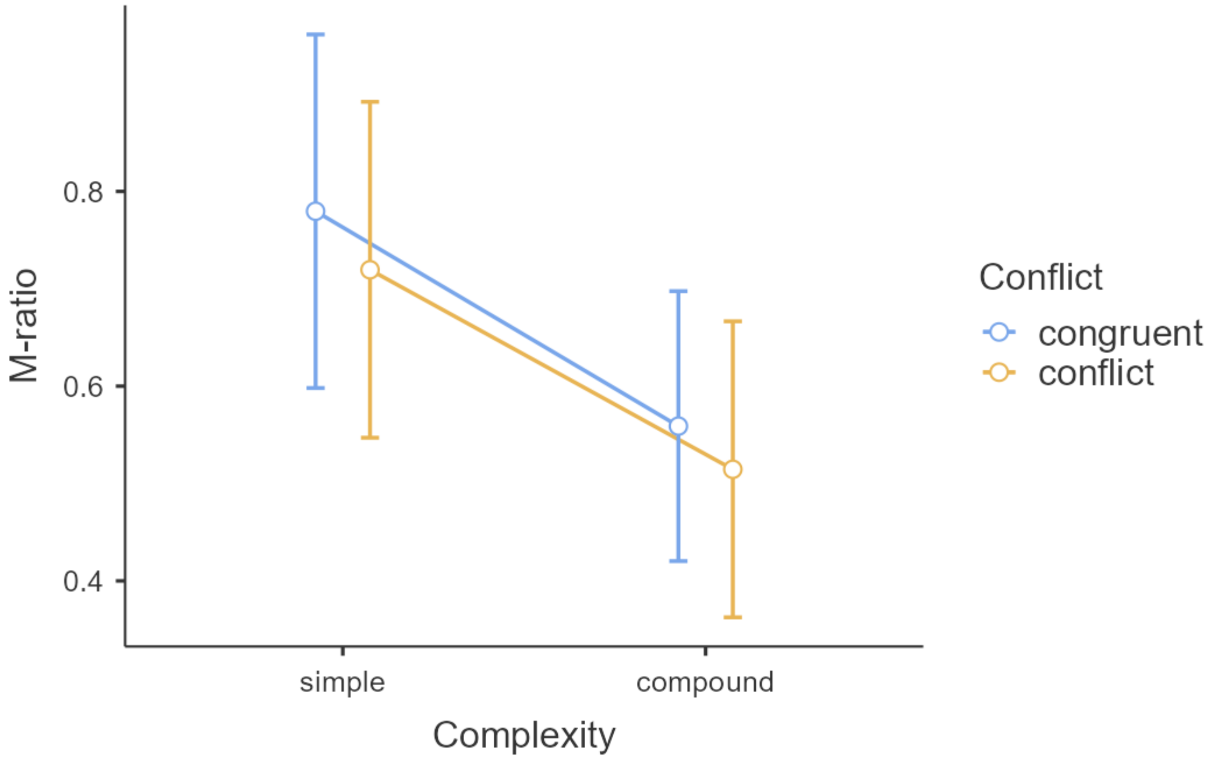

Figure S2. Estimated marginal means of metacognitive efficiency (M-ratio) as a function of Conflict and Complexity in Experiment 2.
